# Interpretation of HDX Data by Maximum-Entropy Reweighting of Simulated Structural Ensembles

**DOI:** 10.1016/j.bpj.2020.02.005

**Published:** 2020-02-15

**Authors:** Richard T. Bradshaw, Fabrizio Marinelli, José D. Faraldo-Gómez, Lucy R. Forrest

**Affiliations:** 1 Computational Structural Biology Section, National Institute of Neurological Disorders and Stroke, National Institutes of Health, Bethesda, Maryland; 2 Theoretical Molecular Biophysics Unit, National Heart, Lung, and Blood Institute, National Institutes of Health, Bethesda, Maryland

## Abstract

Hydrogen-deuterium exchange combined with mass spectrometry (HDX-MS) is a widely applied biophysical technique that probes the structure and dynamics of biomolecules without the need for site-directed modifications or bio-orthogonal labels. The mechanistic interpretation of HDX data, however, is often qualitative and subjective, owing to a lack of quantitative methods to rigorously translate observed deuteration levels into atomistic structural information. To help address this problem, we have developed a methodology to generate structural ensembles that faithfully reproduce HDX-MS measurements. In this approach, an ensemble of protein conformations is first generated, typically using molecular dynamics simulations. A maximum-entropy bias is then applied post hoc to the resulting ensemble such that averaged peptide-deuteration levels, as predicted by an empirical model, agree with target values within a given level of uncertainty. We evaluate this approach, referred to as HDX ensemble reweighting (HDXer), for artificial target data reflecting the two major conformational states of a binding protein. We demonstrate that the information provided by HDX-MS experiments and by the model of exchange are sufficient to recover correctly weighted structural ensembles from simulations, even when the relevant conformations are rarely observed. Degrading the information content of the target data—e.g., by reducing sequence coverage, by averaging exchange levels over longer peptide segments, or by incorporating different sources of uncertainty—reduces the structural accuracy of the reweighted ensemble but still allows for useful insights into the distinctive structural features reflected by the target data. Finally, we describe a quantitative metric to rank candidate structural ensembles according to their correspondence with target data and illustrate the use of HDXer to describe changes in the conformational ensemble of the membrane protein LeuT. In summary, HDXer is designed to facilitate objective structural interpretations of HDX-MS data and to inform experimental approaches and further developments of theoretical exchange models.

## Significance

Hydrogen-deuterium exchange combined with mass spectrometry (HDX-MS) experiments are a powerful approach for probing the conformational dynamics and mechanisms of proteins. However, the mechanistic implications of HDX-MS observations are frequently difficult to interpret because of the limited spatial resolution of the technique, as well as the lack of quantitative tools to translate measured data into structural information. To overcome these problems, we have developed a computational approach to construct structural ensembles that are maximally diverse while reproducing target experimental HDX-MS data within a given level of uncertainty. Using both artificial and experimental test data, we demonstrate that the approach can correctly discern distinct structural ensembles reflected in the target data and thereby facilitate statistically robust evaluations of competing mechanistic interpretations of HDX-MS experiments.

## Introduction

Upon exposure to a deuterated solvent such as D_2_O, labile hydrogen atoms present in protein side chains and backbones will readily exchange for deuterium. The rate of this process is influenced by the chemical features of the exchanging groups and by conditions such as pD or temperature and is also critically dependent on protein conformation (1,2). Consequently, measurements of hydrogen-deuterium exchange (HDX) rates are increasingly used as a direct probe of protein dynamics. Moreover, by combining HDX with mass spectrometry (HDX-MS), this approach has also become feasible for large complexes and membrane proteins, even at low concentrations (3).

Typically, HDX-MS is carried out using so-called bottom-up and continuous labeling strategies, in which proteins are deuterated for varying amounts of time, quenched, proteolytically fragmented, and purified in the solution phase before analysis of the individual peptide fragments by mass spectrometry. For each identified fragment, typically 5–20 residues in length, deuterium incorporation is then reported as the change in peptide mass over time. Because side-chain and terminal-amine deuterons exchange back relatively rapidly with protons during analysis, HDX-MS data reports exclusively on backbone-amide exchange. This ability to directly probe protein dynamics has led to diverse applications (4), including studies of allostery (5, 6, 7), epitope mapping for protein-protein or protein-lipid interactions (8, 9, 10, 11), effects of ligand binding (12, 13, 14, 15), mechanisms of membrane proteins (16, 17, 18, 19, 20, 21, 22), and dynamics of large macromolecular complexes (23, 24, 25, 26). This progress notwithstanding, the interpretation of HDX-MS data in structural and mechanistic terms has been, generally speaking, largely qualitative and lacking objective metrics.

No matter the protein system, interpretation of HDX-MS data requires an understanding of the processes reflected in the exchange kinetics. For any given backbone amide under a given set of conditions (pH, temperature, etc.), the most rapid rate of exchange occurs when the residue is in a completely unstructured, solvent-accessible conformational state of the protein. Under these circumstances, the value of the intrinsic exchange rate constant, $k_{i}^{\mathrm{int}}$ for residue *i*, is determined predominantly by steric and electronic effects from neighboring side chains (27,28). In a folded conformational state, by contrast, amides will be partially or fully occluded from solvent and/or engaged in hydrogen bonding. This structural protection can diminish the intrinsic rate constant by several orders of magnitude. In this case, exchange is better described as a two-step process: first, a structural transition must occur from a so-called noncompetent exchange state to a competent one; this step is followed by the intrinsic chemical exchange reaction with rate constant $k_{i}^{\mathrm{int}}$ (2,29). If the structural transition entails only local alterations rather than complete unfolding, an equilibrium between the exchange-competent and noncompetent states may be reached rapidly, even more so than the hydrogen-deuterium substitution; this situation is referred to as occurring with “EX2” kinetics. The overall exchange rate under these conditions is thus given by the product of the equilibrium constant for the structural transition and the intrinsic rate, *k*_*i*_ = $K_{i}^{\mathrm{eq}}k_{i}^{\mathrm{int}}$. This relationship is commonly expressed as *k*_*i*_ = $k_{i}^{\mathrm{int}}$/*P*_*i*_, where *P*_*i*_ denotes the “protection factor” for each amide, which in turn relates to the free-energy difference between the noncompetent and competent states, *ΔG* = *RT*ln*P*_*i*_. Following these concepts, HDX data is commonly interpreted in terms of the degree of protein structural flexibility and solvent accessibility for a given amide.

In practice, HDX-MS experiments measure deuteration averaged over lengthy peptide fragments rather than at the single-residue level. Even in the light of statistical analysis approaches that allow high-resolution protection factors to be derived directly from experimental data for peptides (30,31), interpretation of the observed data in structural terms is not straightforward. Oftentimes, HDX levels are color coded and mapped on known protein structures, which allows an intuitive visualization of the results and highlights dynamic or solvent-exposed protein regions. However, this kind of qualitative visual analysis can easily lead to a subjective interpretation of the experimental data (32). Moreover, HDX data reflect the properties of an ensemble of protein conformations and, in some cases, therefore might not be explained by a single structural state. To address these issues, previous studies have relied on molecular simulation methods. A typical approach is to first generate a conformational ensemble for the protein of interest with molecular dynamics (MD) or Monte Carlo simulations. The simulated data must then be translated into predicted peptide-deuteration levels that can be correlated with the experimental data (33, 34, 35, 36, 37, 38, 39, 40, 41, 42, 43). Typically, this is achieved using empirical models that predict protection factors *P*_*i*_ from an ensemble of protein structures. Some *P*_*i*_ prediction models directly estimate the free energy of exchange from the equilibrium constant obtained by defining both exchange-competent and noncompetent states from a simulated trajectory (34,36,40), which requires that both sets of states have been adequately sampled. In a second category of *P*_*i*_ prediction models, such as the one used here (33), the likelihood of exchange is predicted using an empirical scoring function parameterized on the basis of the characteristics of the folded state only; thus, *P*_*i*_ may be in principle predicted from sufficiently long (e.g., microsecond-timescale) MD simulations of one or more folded states of the protein of interest. Regardless of the specific approach, an important caveat of these kind of strategies is that for many cases of interest, a simulation may not accurately represent the conformational ensemble reflected by the experimental data, for example, because of force-field inaccuracies or incomplete sampling of alternate folded states. Thus, even if a perfectly accurate empirical model for *P*_*i*_ were at hand, the predicted protection factors might deviate substantially from measured data.

Here, we develop and test a methodology to resolve this problem. This approach, which we refer to as HDX ensemble reweighting (HDXer), enables us to construct conformational ensembles that faithfully reflect a given set of target HDX-MS data for a given empirical model of *P*_*i*_. HDXer is based on concepts outlined in previous studies and applied to other types of biophysical data (44, 45, 46, 47, 48, 49, 50, 51, 52, 53, 54) but not yet to HDX-MS. In brief, this is a post hoc method whereby a maximum-entropy criterion is used to reassign statistical weights to each of the configurations in a structural ensemble generated computationally (e.g., via simulations or modeling) so that calculated ensemble-averaged peptide deuterated fractions reproduce measured values within a given level of uncertainty. That is, this approach aims to adjust populations in a heterogenous conformational ensemble such that they conform ideally to the experimental data while taking into account all potential sources of uncertainty. Thus, the method can be used to rank the correspondence between a given HDX-MS data set and several candidate conformational states based on the degree of bias required to reproduce the experimental results.

To evaluate the validity of HDXer, we focus primarily on artificial HDX-MS data generated for a binding protein that undergoes a substantial conformational change (55). Specifically, we reweight a simulated structural ensemble so that calculated deuteration levels match a set of artificial HDX-MS data reflecting predefined populations of two major conformational states. The performance of the method is then assessed based on whether the conformations favored by the reweighting indeed correspond to the structural states used to generate the target data. The use of artificial data allows us first, to rigorously assess reweighting performance in a context for which the correct ensemble is known and second, to evaluate the effect of different sources of uncertainty on the ensemble reweighting. Encouragingly, the results show that the proposed approach always recovers the key features of the correct structural ensemble, even when sparse HDX data are targeted or in the presence of moderate error sources. Finally, to demonstrate the transferability of the approach to experimentally determined data, we apply HDXer to HDX-MS measurements obtained recently for the membrane transporter LeuT (16).

## Methods

### Calculation of HDX residue protection factors and peptide deuterated fractions

To predict deuterium uptake based on structural snapshots (obtained from MD simulations or another molecular modeling method), we first calculate the protection factor for each residue *i*, *P*_*i*_, using the method of Best and Vendruscolo (33). Specifically, the free-energy difference between exchange-competent and noncompetent states of a residue is approximated by a linear function of the numbers of H-bonds and heavy-atom contacts of the corresponding backbone amide, denoted as *N*_H,__*i*_ and *N*_C,__*i*_, respectively:

(1) $$\ln\;P_{i}=\langle \beta_{C}N_{C,i}+\beta_{H}N_{H,i\;}\rangle .$$

The notation 〈…〉 signifies an ensemble average over all available snapshots. *N*_C,__*i*_ is calculated as the number of nonhydrogen atoms within 6.5 Å of the amide N atom of residue *i*, excluding atoms in residues *i* − 2 to *i* + 2; *N*_H,__*i*_ is the number of O or N atoms within 2.4 Å of the amide hydrogen atom. In the original formulation by Best and Vendruscolo, the scaling factors *β*_C_ and *β*_H_ are set to 0.35 and 2.0, respectively. These values reflect an empirical optimization with respect to experimental HDX data for several water-soluble proteins (33); however, their optimal value depends on the protein or experimental conditions (43), and therefore, we will treat them as optimizable parameters.

In addition to *P*_*i*_, we consider the intrinsic exchange rate constant for each residue type, $k_{i}^{\mathrm{int}}$, from Bai and co-workers, updated for acidic residues and glycine (27,28). Deuterated fractions for peptide segments of the protein, $D_{j,t}^{\mathrm{sim}}$, can then be calculated for any given time point of exchange, *t*, using the exchange rate constants of each individual residue and according to first-order kinetics. That is,

(2) $$D_{j,t}^{\mathrm{sim}}=\frac{\sum_{i=m_{j}+1}^{i=n_{j}}1-\text{exp}\left(-\frac{k_{i}^{\mathrm{int}}}{P_{i}}t\right)}{n_{j}-m_{j}},$$

where *m*_*j*_ and *n*_*j*_ are the first and last residue numbers of the *j*-th protein fragment respectively. Note that proline residues do not have an exchangeable amide proton and were therefore excluded from the deuterated fraction calculation. The first residue (*m*_*j*_) in each peptide segment was also omitted from the average because hydrogens in the amine N-terminus are labile after proteolytic fragmentation and are assumed to have fully exchanged back to protons during the HDX-MS purification and analysis step. It should also be noted that in direct comparisons of experimental and predicted data, the measured deuterated fractions should be corrected for the fraction of D_2_O/H_2_O in the reaction buffer and for back exchange during the analysis process. Both corrections can be achieved by normalizing to deuterated fractions observed in identical control experiments performed under maximal deuteration conditions (32).

### Maximum-entropy ensemble reweighting with HDX data

In this section, we describe the basic formulation for calculating corrections to the statistical weight of the individual structural snapshots in an ensemble, each denoted by *X*_*k*_, such that the predicted deuteration fractions reproduce a set of HDX experimental data. Our approach is related to that of Marinelli and Fiorin (46), in which the only bias applied is that strictly required to conform to the experiments, following the so-called maximum-entropy principle (44,45,53,54,56). In general terms, the minimal bias needed to correct the mean value of one or more observables of interest is provided by a linear function of those observables, added as a perturbation term to the molecular force field or energy function, *U*(*X*) (44). In this case, the target observables are *P*_*i*_ (or functions thereof) (Eqs. 1 and 2), and therefore the corrected force field is defined as

(3) $$U_{\mathrm{corr}}\left(X\right)=U\left(X\right)-k_{B}T\sum_{i}\lambda_{i}\left[\beta_{C}N_{C,i}\left(X\right)+\beta_{H}N_{H,i}\left(X\right)\right].$$

In the initial sample, the statistical weight of each configuration *X*_*k*_ is proportional to *exp*{−*U*(*X*_*k*_)/*k*_B_*T*}. Similarly, in the corrected ensemble, these weights are proportional to *exp*{-*U*_corr_(*X_k_*)/*k*_B_*T*}. The set of weight adjustments we seek, *Ω*(*X*_*k*_), are therefore simply a Boltzmann factor of the linear term of Eq. 3:

(4) $$\Omega \left(X_{k}\right)=\frac{exp\left\{\sum_{i}\lambda_{i}\left[\beta_{C}N_{C,i}\left(X_{k}\right)+\beta_{H}N_{H,i}\left(X_{k}\right)\right]\right\}}{\sum_{k^{\prime }}exp\left\{\sum_{i}\lambda_{i}\left[\beta_{C}N_{C,i}\left(X_{k^{\prime }}\right)+\beta_{H}N_{H,i}\left(X_{k^{\prime }}\right)\right]\right\}},$$

in which the denominator is a normalization term calculated by summing over all simulation configurations.

The scaling factors *λ*_*i*_ in Eqs. 3 and 4 are the key adjustable parameters in this methodology. These parameters will be uniquely determined so that deuteration fractions deduced from the reweighted ensemble fit the experimental data within a defined error distribution, *ρ*_err_, and with the smallest possible bias. To quantify this bias, we report the amount of apparent work, *W*_app_, required to reweight the ensemble. In formal terms, the optimal value of *λ*_*i*_ is at the global minimum of the following (Kullback-Leibler) likelihood function (46,57):

(5) $$L=\frac{W_{\mathrm{app}}}{k_{B}T}-\ln\rho_{\mathrm{err}}.$$

The apparent work, *W*_app_, depends on the correction to the potential applied in Eq. 3 as follows:

(6) $$W_{\mathrm{app}}=k_{\text{B}}T\text{ ln}\langle \text{exp}\left\{-\sum_{i}\lambda_{i}\left[\beta_{C}N_{C,i}\left(X\right)+\beta_{H}N_{H,i}\left(X\right)-lnP_{i}\right]\right\}\;\rangle ,$$

where 〈…〉 denotes a mean value over the corrected ensemble or, in other words, a weighted average according to the weights of Eq. 4. Note that *W*_app_ is related to the Kullback-Leibler divergence between the initial and corrected ensembles (46,57,58), *d*_KL_ = *W*_app_/*k*_B_*T* = $\sum_{k}\Omega \left(X_{k}\right)$ln*Ω*(*X*_*k*_) + ln*N*, where *N* is the number of simulation frames.

The function *ρ*_err_ is an error distribution that, for simplicity, we assume to be Gaussian and uncorrelated across all target data points:

(7) $$\rho_{\mathrm{err}}\left(D^{\mathrm{sim}}\right)\propto \exp\left\{-\sum_{t}\sum_{j}\gamma \frac{{\left(D_{j,t}^{\mathrm{sim}}-D_{j,t}^{\exp}\right)}^{2}}{2\eta^{2}}\right\},$$

where the parameter *γ* controls the final level of agreement with the target experimental data (see below), *η* is an estimate of the uncertainty (here set to 1, such that *γ* instead imposes equal uncertainty for all target data points), and $D_{j,t}^{\exp}$ and $D_{j,t}^{\mathrm{sim}}$ are the experimental and predicted deuterated fractions, respectively. $D_{j,t}^{\mathrm{sim}}$ is calculated according to Eq. 2 using the protection factors for each amide, but after adjusting for reweighting, $\ln\;P_{i}=\langle \beta_{C}N_{C,i}+\beta_{H}N_{H,i}\rangle =\sum_{k}\left[\beta_{C}N_{C,i}\left(X_{k}\right)+\beta_{H}N_{H,i}\left(X_{k}\right)\right]\Omega \left(X_{k}\right)$.

In practice, we use a gradient-based minimization of the likelihood function *L* in Eq. 5, in which the parameters *λ*_*i*_ are calculated iteratively according to the derivative of *L*:

(8) $$\lambda_{i}^{n+1}=\lambda_{i}^{n}\left(1-\varepsilon \right)+\varepsilon \frac{\partial \;\ln\rho_{\mathrm{err}}\left(D^{\mathrm{sim}}\right)}{\partial \;\ln P_{i}},$$

where *ε* is an update rate selected to ensure convergence. The corrected (reweighted) protection factors entered into Eq. 8 depend on *λ*_*i*_ (Eq. 4) and thus are also updated at each iteration. The model parameters *β*_C_ and *β*_H_ are optimized at each step using a Monte Carlo procedure to reduce the discrepancy between simulated and experimental data, measured by the mean squared deviation, *MSD* = *χ*^2^/*N*_D_, where *N*_D_ is the number of data points and $\chi^{2}=\sum_{t}\sum_{j}{\left(D_{j,t}^{\mathrm{sim}}-D_{j,t}^{\exp}\right)}^{2}/\eta^{2}$. Optimization was performed with 100 Monte Carlo trials at each step, and new values of *β*_C_ and *β*_H_ were accepted only if *MSD* decreased. The maximum step sizes for the trials (*Δβ*_C,max_ = 0.15, *Δβ*_H,max_ = 1.6) were chosen to correspond to 10% of the maximum range of *β*_C_ and *β*_H_ we proposed to explore.

We note that although HDX-MS measures the total deuterated fraction for protein fragments, our approach uses the minimal bias condition to spread such experimental information across individual residues (Eqs. 3, 4, 5, 6, 7, and 8). Nevertheless, if multiple experimental data points incorporating a single amide are available or if deuteration is otherwise correlated between amides, the contribution of each amide to the ensemble correction is constrained by a simultaneous fit to all the experimental data. Therefore, in practical applications of reweighting, the inclusion of HDX-MS measurements for overlapping peptide segments will ultimately lead to enhanced resolution.

### Reweighting parameters and metrics of robustness

In the reweighting procedure, the presence of unknown errors in predicted and experimental data is implicitly considered by setting a parameter *γ* (Eq. 7) that regulates the variance in the error distribution and that can be tuned to achieve a compromise between the applied bias and the level of agreement with experiments (46,57). To identify a reasonable value of *γ*, a decision plot of *W*_app_ vs. *MSD* can be constructed for different values of *γ*. Typically, the presence of undetermined, systematic errors such as forward-model uncertainty or sampling inefficiency induces a rapid increase of the work value below a certain value of *MSD*, resulting in an L-shaped decision plot (see Fig. 6 *A* for an example). In this case, a reasonable value of *γ* can be found at the kink of the L-curve, provided that the associated value of *W*_app_ is within say, two or three *k*_B_*T*.

### TeaA simulation data and generation of the artificial target HDX data

**Figure 1:**
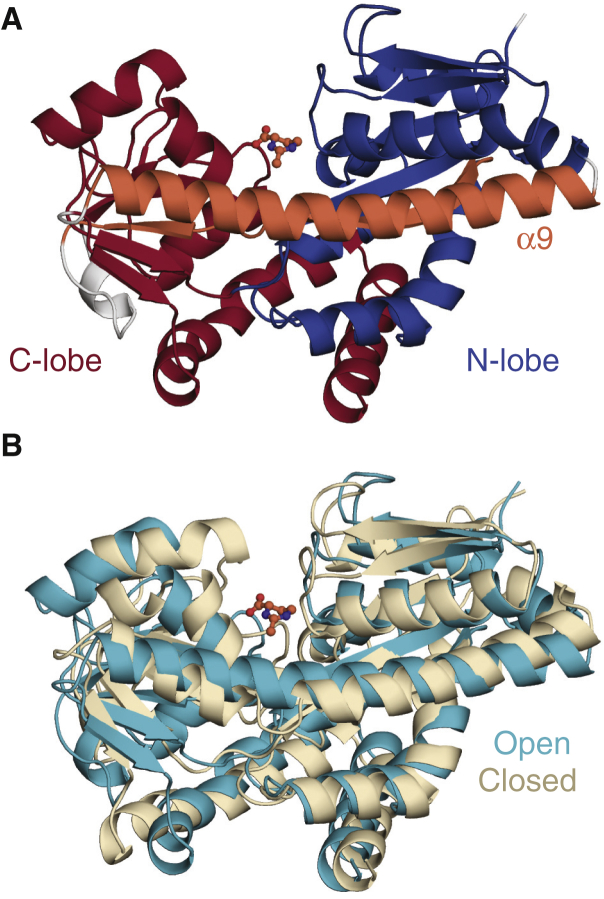
Structures of ectoine-bound TeaA in open and closed conformations. (*A*) A representative open structure is shown as cartoon helices, highlighting the N-lobe (*blue*), the C-lobe (*red*), and the *β*4/*α*9 segments (*orange*) that span both lobes. The ectoine ligand bound to the central binding cleft is shown in ball-and-stick representation. (*B*) An overlay of representative open (*cyan*) and closed (*wheat*) conformations is given. The C_*α*_ RMSD between the two conformations is 3.2 Å.

The simulation data used for ensemble reweighting were taken from the unbiased replica (∼45 ns, with frames at 1 ps intervals) of bias-exchange metadynamics simulations performed previously (55) for the periplasmic binding protein TeaA from *Halomonas elongata* (UniProt: E1VBK1), including both “closed” and “open” states of TeaA (Fig. 1). The artificial HDX-MS data used as a target for the reweighting were created from this trajectory so as to represent a rapidly interconverting conformational ensemble comprising 60% closed and 40% open states. Specifically, two reference configurations were chosen to represent typical “closed” and “open” states based on their structural similarity to available structures (Fig. 1 *B*), and two subensembles of closed and open configurations (corresponding to 37.2 and 1.6% of the initial frames, respectively) were then obtained by extracting highly related frames, defined as those in which the root mean-square deviation (RMSD) of the C_*α*_ atoms was <1.0 Å from those in the closed or open reference structures. The remaining 61.2% of frames remained unassigned.

Artificial target HDX-MS data sets were then derived from the closed and open subensembles according to Eq. 2. Residue protection factors for the mixed target ensemble were calculated as $\ln\;P_{i}^{\mathrm{mix}}=0.6\;\ln\;P_{i}^{\mathrm{closed}}+0.4\;\ln\;P_{i}^{\mathrm{open}}$, in which $P_{i}^{\mathrm{closed}}$ and $P_{i}^{\mathrm{open}}$ represent protection factors calculated across the subensemble of the closed and open conformations, respectively. Protection factors were calculated using Eq. 1, with *β*_H_ = 2.0 and *β*_C_ = 0.35. To assess the ability of our reweighting method to extract subensembles of different relative size, additional target ensemble mixtures were created with 95:5, 80:20, 40:60, 20:80, and 5:95 ratios of closed/open conformations. Artificial HDX-MS data were also constructed for the open and closed ensembles separately using the same values of $P_{i}^{\mathrm{closed}}$ and $P_{i}^{\mathrm{open}}$. All artificial data were calculated at time points of 0.167, 1.0, 10.0, 60.0, and 120.0 min. These time points reflect typical HDX-MS experiments and capture both short- and long-timescale EX2 exchange. A scheme of the generation and use of the artificial mixed-ensemble HDX data for ensemble refinement is provided in Fig. S1.

To assess the impact of segment averaging and sequence coverage upon reweighting, multiple TeaA HDX-MS data sets were generated. The largest set of artificial HDX measurements, obtained at residue-level resolution and with full sequence coverage, comprised 294 residues at five time points, giving a total of 1470 individual predicted observables to be refined against. To evaluate the effect of segment averaging, five other target data sets were generated, in which TeaA was divided into fragments of size 5, 10, 15, 20, or 50 residues, including prolines. Note that because deuteration of the N-terminal amine is excluded from HDX-MS data, neighboring protein segments were defined with a one-residue overlap (e.g., 1–10, 10–19, etc.). The final peptide segment in each data set was extended up to the C-terminal residue 310. Analysis of the effect of sequence coverage was based on the 10-residue segment target HDX data set, which comprises 34 peptides, from which coverage was reduced in five cumulative steps from 100 to 20% of the sequence (six to seven peptides at each step; Fig. S2). Assuming that buried peptides are less likely to be proteolytically hydrolyzed, we preferentially excluded peptides with lower solvent accessibility.

To assess the effect of experimental noise on the reweighting, we added an error term to each target HDX data point; the magnitude of this error was randomly obtained from Gaussian distributions of standard deviation *σ* = 0.01 or 0.1 (in units of deuterated fraction). The target HDX-MS data in this case were those generated to reflect 100% protein coverage and 10-residue segments.

To evaluate the impact of sampling errors, we removed from the simulation data all frames with C_*α*_ RMSD < 1.5 Å with respect to the reference closed-state structure.

To assess the effect of the accuracy of the model used to predict protection factors (Eq. 1) on the ensemble reweighting, we modified the value of the *β* parameters in different protein regions (as opposed to uniform values of *β*). Two different target HDX data sets were generated, both containing variations in the model parameters for residues 225–261, which comprise the *α*9 helix. Specifically, the *β* parameters were selected to produce “low-error” (*β*_H_ = 7.0, *β*_C_ = 0.2) and “high-error” (*β*_H_ = 2.0, *β*_C_ = 0.2) target data sets using values that are either consistent or inconsistent, respectively, with their observed inverse relationship (see Fig. S8).

### Trajectory clustering

To interrogate the results of the reweighting procedure without the requirement for a reference protein configuration, the structures (“samples”) in the final ensembles were clustered based on their pairwise similarity (RMSD of the C_*α*_ traces) using the density-based algorithm DBSCAN as implemented in scikit-learn v0.21.2 (59). The minimum size of a cluster, *n*, was set to 10% of the total ensemble size, but the contribution of each frame to *n* corresponded to the weight assigned after ensemble reweighting (Eq. 4) and normalized to the number of structures in the entire ensemble. The maximum radius, *ε*, which defines the neighborhood of an individual sample, was chosen by evaluating cluster quality for the ensemble obtained after reweighting to the residue-level data set with *γ* = 10^3^. Scanning a range of values of *ε* from 10.546 to 105.46 Å (equivalent to pairwise RMSD values of 0.05 or 0.50 Å, respectively) on this test set revealed well-defined clusters with high silhouette scores (60) at an *ε*-value of 42.187 Å (a pairwise RMSD of 0.20 Å).

### Application of HDXer to LeuT HDX-MS data

We considered the experimental HDX-MS data for the amino acid transporter LeuT (UniProt: O67854) described in a previous study by Adhikary et al. (16). Briefly, these data had been obtained at 0.167, 1.0, 10.0, and 120.0 min time points for wild-type (WT) and Y268A mutant LeuT reconstituted into 60/40 1-palmitoyl-2-oleoylphosphocholine (POPC)/1-palmitoyl-2-oleoylphosphoglycerol (POPG) lipid nanodiscs in 20 mM Tris-HCl (pH 7.4), 100 mM NaCl, 0.5 mM EDTA buffer. Of the 21 peptides previously identified and compared (16), 17 peptides were used for HDXer analysis. The other four peptides, in either the N-terminus (1–12, 1–14, 1–16) or the C-terminus (505–517), were not used because they are found in regions not resolved in the crystal structures used in our MD simulations.

Reference structural ensembles of WT and Y268A LeuT were generated by extending the atomistic MD simulations performed by Adhikary et al. (16). Briefly, structures of either outward-facing WT LeuT (starting from Protein Data Bank, PDB: 3TT1) or inward-facing Y268A LeuT (starting from PDB: 3TT3) were embedded in a 1,2-dimyristoylphosphocholine (DMPC) bilayer, and three independent, 2-*μ*s-long simulations were performed for each configuration. Structures were saved at 100 ps intervals for a total of 20,000 frames per 2 *μ*s simulation, and hence, a total of 120,000 frames in the reference ensemble were used as input to HDXer.

### Data availability

All underlying data used in this study are made freely available (https://doi.org/10.5281/zenodo.3385168), including the initial simulation trajectories, target HDX data sets, and analysis code for extracting contacts and H-bonds, generating artificial target data sets, reweighting ensembles, and clustering. The code and underlying data used to create figures are also available in this repository.

## Results

### The TeaA test system undergoes a substantial conformational change

In the proposed computational approach, we seek to be able to reweight a heterogenous structural ensemble so that it optimally reflects a given set of HDX-MS data. The success of such a method requires that it be able to detect and upweight the protein configurations that are most consistent with the data but also to detect and downweight those that are not. To meaningfully test this method, therefore, one must begin with a sample that is sufficiently heterogeneous for a system with several states of known structure. To this end, we considered the ectoine-binding protein TeaA and extracted a broad sample of configurations from enhanced-sampling MD simulations carried out in a previous study (55). The structure of TeaA consists of two distinct lobes interconnected by a single *β*-strand (*β*4) and a single *α*-helix (*α*9) (Fig. 1 *A*). Ectoine binding at a central cleft between the lobes fosters a clamshell-like structural change (Video S1), whereby the distance between lobes changes by up to ∼10 Å. We refer to the two endpoints of this conformational change as the “open” and “closed” states (Fig. 1 *B*). These states have nearly identical secondary structure, except that closure requires local unwinding and kinking of the *α*9 helix at residues K247-L249.

Video S1. Artificially Generated Morph between the Closed and Open Representative Structures of TeaA TeaA is shown in cartoon representation (*wheat*), and the ectoine substrate from the closed configuration is shown in ball and stick representation (*peach*).

The existing simulations, based on bias-exchange metadynamics, capture the full range of this structural change and explain how the affinity for ectoine is modulated by the conformational state of the protein (55). These data demonstrated that the closed state of TeaA is most favored when ectoine is bound; however, partial opening of this bound form was also observed and found to entail a free-energy penalty of only ∼2 kcal mol^−1^ (55). Accordingly, the unbiased replica in these simulations samples open, closed, and intermediate configurations of the protein (Fig. S3). This structural heterogeneity makes these data an ideal choice as a reference set on which to test the performance of our reweighting method.

### Artificial HDX-MS data for open and closed TeaA

To test the protocol proposed here, we also need target HDX data sets for each of the conformation states of the protein of interest. To our knowledge, however, no experimental HDX-MS data exist for TeaA. We therefore decided to generate artificial, high-resolution HDX data for the two major states of TeaA (open and closed) to evaluate whether a hypothetical experiment would yield a measurable contrast. To this end, we extracted separate ensembles of open and closed conformations from the simulation data and compared the predicted deuterated fractions at the single-residue level for each set (calculated using Eqs. 1 and 2). The HDX data were generated at single-residue resolution and across five time points to capture both spatial and temporal differences in deuterium uptake at high resolution (see Methods).

**Figure 2:**
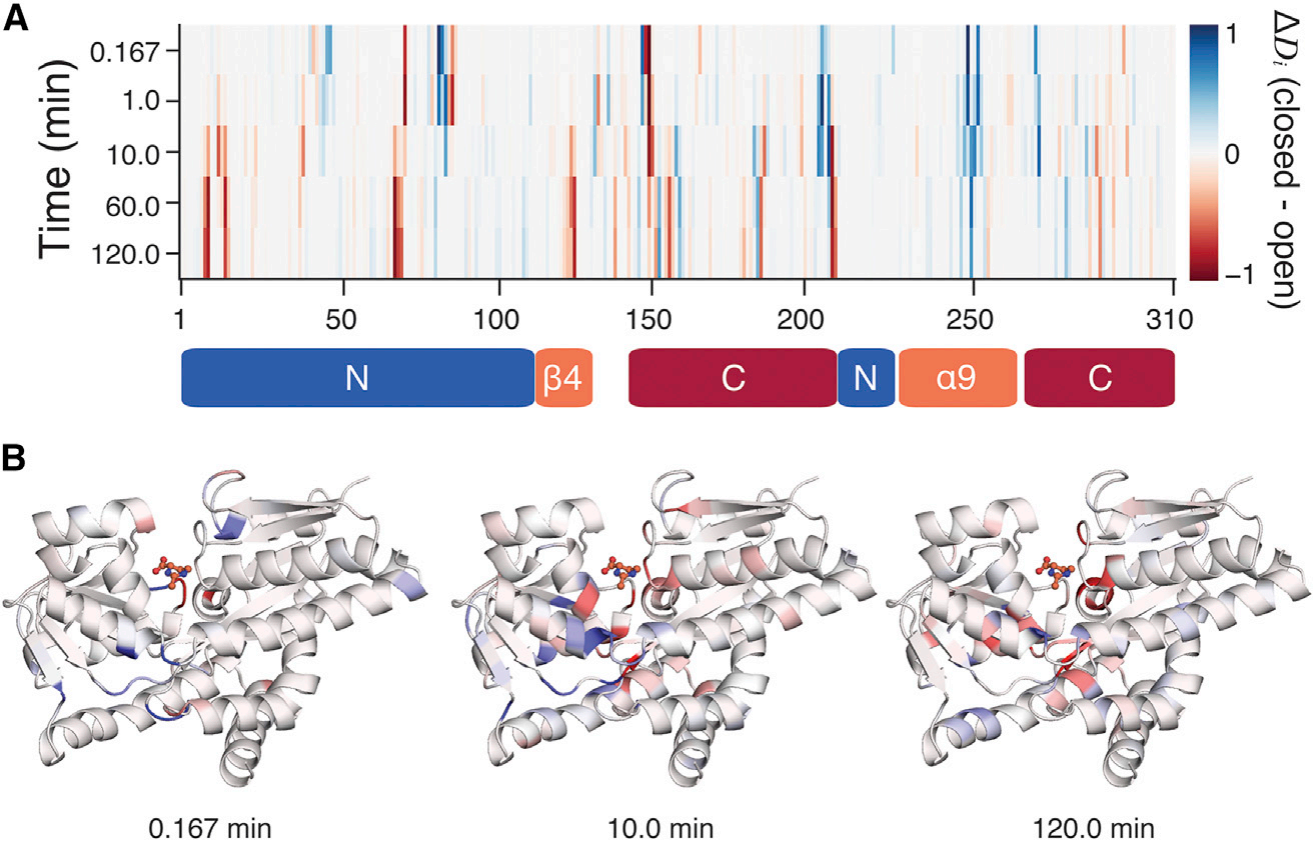
Difference in predicted deuterated fractions between closed and open ensembles of TeaA. (*A*) By-residue *ΔD*_*i*_ = *D*_*i*,closed_ − *D*_*i*,open_ for each time point is shown, where red indicates that a residue is more deuterated in the open conformation than in the closed, and blue indicates the opposite. Domain definitions are indicated using bars beneath the plot. (*B*) A representative closed structure of TeaA, colored by residue *ΔD*_*i*_ at the 0.167, 10, and 120 min time points, is shown. The largest *ΔD*_*i*_-values are observed for residues either lining the central binding cleft or involved in the partial unfolding of helix *α*9 but are clearly not uniform across time points.

We observed substantial differences between the predicted deuterated fractions of closed and open ensembles (Fig. 2 *A*), confirming that these artificial data sets are well suited for our purpose. As might be expected, this contrast is most pronounced at the binding site interface and in the *α*9 helix (Fig. 2 *B*). Interestingly, though, subtle differences are also observed across almost the entire protein and vary from one time point to another. These complex patterns cannot be easily interpreted visually, e.g., by mapping the data onto the representative structures (Fig. 2 *B*) because they reflect the dynamical nature of the simulated ensembles. For the same reasons, such comparisons based on single structures also offer limited insights into experimentally determined HDX-MS data, as has been noted elsewhere (32). The striking variability of the idealized artificial data for TeaA further illustrates the need for an ensemble perspective to rigorously interpret HDX measurements at the structural level.

### Ensemble reweighting with idealized single-residue HDX target data

**Figure 3:**
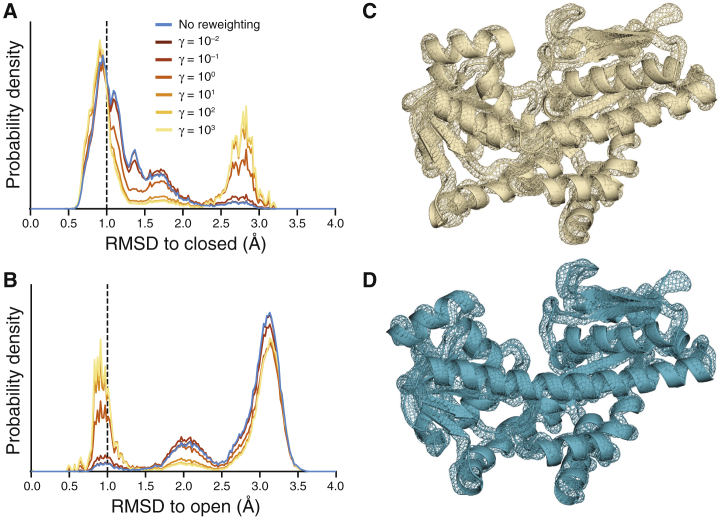
Results of HDX ensemble reweighting at single-residue resolution. (*A* and *B*) Probability distributions of the RMSD are shown with respect to the closed (*A*) or open (*B*) reference structure of TeaA for the initial reference ensemble (*cyan*) and for ensembles obtained after reweighting with progressively higher *γ*-values (*dark brown* to *orange* to *yellow*). The dashed line indicates the 1.0 Å RMSD cutoff used to assign frames to the reference closed (*A*) or open (*B*) ensemble. (*C* and *D*) Ensemble density maps of the closed (*C*) or open (*D*) clusters, extracted by structural clustering after reweighting with *γ* = 10^3^, are given. The mesh reflects the density of backbone N, CA, and C atoms overlaid onto the representative closed (*C*) or open (*D*) structure of TeaA. Maps were created using the AtomProb (75) feature of Xplor-NIH v2.51 and are shown at 0.25 *σ*.

To begin to evaluate the HDXer method, we next produced artificial HDX-MS data for a hypothetical measurement in which TeaA spontaneously interconverts between closed and open states, populating these states in a 60:40 ratio. The sample derived from the unbiased metadynamics replica (hereafter referred to as the reference ensemble) comprises a heterogeneous set of conformations that were either assigned to closed and open states or unassigned (decoys), with a ratio of 37.2:1.6:61.2 (see Methods). Note that some of the decoy structures do share structural similarities with either open or closed states, as demonstrated by the continuity of the RMSD distributions (Fig. 3, *A* and *B*, *cyan*). The challenge for the HDXer method, therefore, is to identify to the appropriate weights for each and all of the configurations in the reference ensemble so that ensemble-averaged HDX levels calculated for the reweighted sample exactly reflect the 60:40 ratio of open/closed conformations in the target data set.

As expected, without reweighting, the predicted HDX levels for the reference ensemble were in poor agreement with the target HDX data (*MSD* = 2.2 × 10^−3^, equivalent to a root mean-square error of 0.047 Da in mass for every residue), owing to the mismatch in populations. In reweighting applications, acceptable levels of *MSD* are highly system and application dependent. With artificial data, because the target ensemble is exactly present in the reference ensemble, we considered *MSD* ≤ 10^−6^, which is equivalent to a root mean-square error of 0.001 Da in mass for every residue, to be an acceptable agreement in this case. Ensemble reweighting with HDXer succeeds in matching the target data (Fig. 3; Fig. S4) to an extremely high level of precision. By increasing the value of the parameter *γ* in Eq. 7, an increasingly tighter agreement with the target HDX data was achieved (Fig. S4 *A*), requiring a larger apparent work, *W*_app_, to be applied (Fig. S4 B) and resulting in an increasing deviation from the initial reference ensemble, whereas the optimized values of *β*_C_ and *β*_H_ remain close to their target values (Fig. S4, *C* and *D*). Enforcing closer agreement with the target HDX (by increasing *γ*) resulted in the gradual development of a RMSD distribution profile containing two distinct peaks, corresponding to the closed and open states of TeaA, as the decoy trajectory frames became downweighted (*dark brown*, *orange*, and *yellow*; Fig. 3, *A* and *B*). Notably, the bimodal features of the target distribution could already be detected with only a small applied bias of *W*_app_ = 0.9 kJ mol^−1^ relative to the 2.6 kJ mol^−1^ bias applied at *γ* = 10^3^. After reaching an *MSD* ≤ 10^−7^ (reweighting with *γ* ≈ 10^2^ or larger), no further substantial changes in the ensemble were observed (Fig. 3, *A* and *B*), and *W*_app_ reached a plateau (Fig. S4 B).

To more quantitatively characterize the outcome of the reweighting, we applied a clustering algorithm to the configurations in the reweighted ensemble obtained using *γ* = 10^3^ (see Methods). Two clusters were found: the largest cluster clearly represented a closed conformation (Fig. 3 *C*) and comprised 59.3% of the final ensemble by weight, whereas the second cluster comprised 35.5% of the final ensemble and reflected an open conformation (Fig. 3 *D*). The remaining 5.2% of the ensemble consisted of outliers that, owing to structural dissimilarities and/or low weight after reweighting, could not be assigned to either of the clusters.

From the RMSD distributions of the reweighted ensembles, it was clear that the final ensemble still contained a non-negligible fraction of frames >1.0 Å RMSD to either the closed or open state. Moreover, these decoy frames were included in the extracted clusters alongside the “correct” frames (i.e., those assigned to the closed or open reference ensembles). These observations raise concerns about the fidelity that can be achieved with ensemble-averaged observables such as these. We therefore asked how similar these decoy structures are to those used to generate the target protection factor data. According to the root mean-square fluctuation of the backbone atoms, both clusters exhibited minimal structural variance, with a maximum root mean-square fluctuation of 1.2 Å, excluding the N-terminal residue (Fig. S5), and well-defined backbone density when calculated across all structures in each cluster (Fig. 3, *C* and *D*). Reassuringly, then, the inclusion of decoy frames reflected conformationally correlated frames, indicating that the reweighting identified key structural features of the target data and, based on those features, created populations of the two conformational states in good agreement with the target ratio of 60:40.

### HDX ensemble reweighting with realistic peptide segments and sequence coverage

The results so far demonstrate that HDX reweighting can successfully extract key structural features of artificial target HDX data for an ensemble of conformations defined at the residue level and with 100% sequence coverage. However, this level of information content is not representative of typical HDX-MS experiments, which report deuterated fractions for proteolytic fragments of a protein, whereas complete sequence coverage requires extensive optimization of experimental conditions. To evaluate the extent to which lower-resolution HDX-MS data can be meaningfully interpreted with a quantitative method such as HDXer, we systematically degraded the information content of the artificial target data produced at single-residue resolution while maintaining the 60:40 ratio of closed/open state data. First, the deuterated fraction values were averaged over peptides of increasing length, from 5 to 50 residues, while maintaining full sequence coverage. Second, using fragment lengths typical for HDX-MS, sequence coverage was reduced by removing peptide segments from the target data. To compare ensembles obtained with different target data sets, for which *γ*-values are not directly comparable, we instead fixed the level of agreement with the target data at *MSD* = 10^−6^.

**Figure 4:**
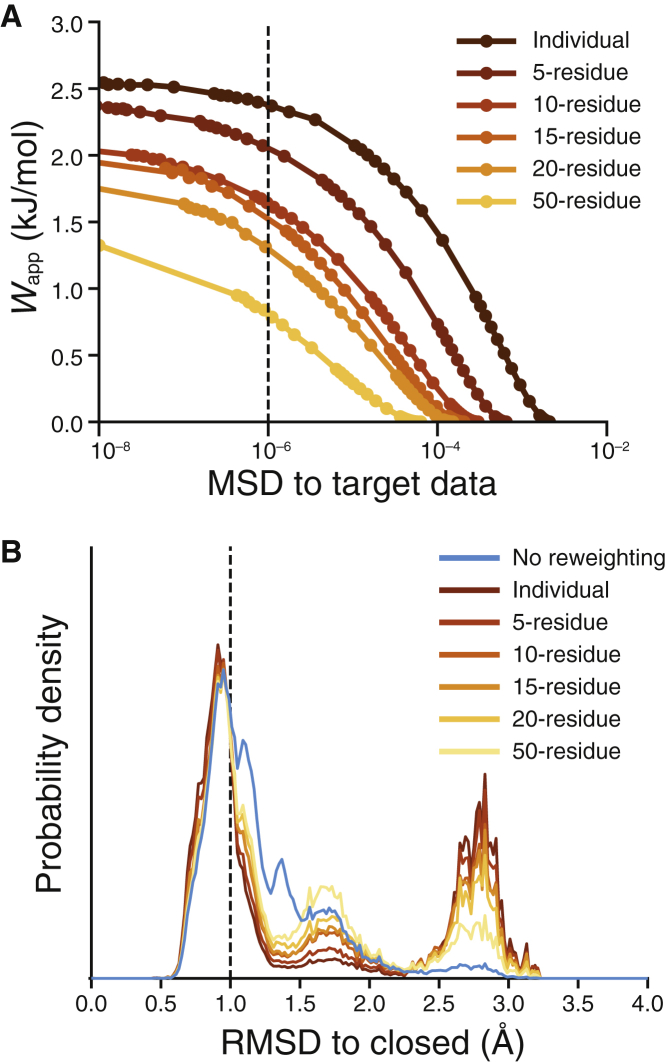
Effects of segment averaging on ensemble reweighting. (*A*) A decision plot is given showing the work applied during reweighting against the *MSD* of the reweighted ensemble to target HDX data. Circles indicate independent reweighting experiments. (*B*) RMSD probability distributions, with respect to the closed TeaA structure, are shown before (*cyan*) and after ensemble refinement to *MSD* = 10^−6^. In both panels, data from reweighting performed with individual residue deuterated fractions are shown in dark brown, and the data obtained by increasing the peptide segment lengths are shown using gradual color variation from light brown to orange to yellow.

Averaging the deuterated fractions over peptide segment lengths from 5 to 50 residues represents a loss of spatial resolution in the HDX-MS signal and increases the degeneracy of the structural information present in the data. When reweighting the reference ensemble, increasing the length of the segments progressively reduced the value of *W*_app_ required to achieve the same level of agreement with the target data, which is also increasingly less resolved and thus more easily reproduced (Fig. 4 *A*). That is, the smaller values of *W*_app_ reflect a greater similarity between the initial and reweighted ensembles. However, this degradation of the target data translates into a reduced ability to discern between conformational states. Specifically, both the RMSD probability distributions and the structure-based clustering after reweighting (Fig. 4 *B*; Table 1) show that increasing the fragment length reduced the ability of HDXer to discriminate between open and semiopen (RMSD ∼1.7 Å) protein structures. Indeed, using ≥20-residue-long segments, the semiopen state was still highly populated and was identified as a separate, unique cluster (Table 1). The decreasing structural fidelity of the results was correlated with larger deviations of the HDXer-optimized *β*_H_ and *β*_C_ parameters. However, optimized parameters remained close to their target values for segments ≤20 residues, in which maximal deviations of *β*_C_ ± 2.2% and *β*_H_ ± 8.5% were observed. For longer fragments, therefore, a quantitative interpretation is not possible, unless information from overlapping (redundant) peptides is available (Fig. S6). Overall, however, for the peptide lengths typical of HDX-MS experiments (5–20 residues), HDX reweighting correctly identified the trends in closed and open state populations present in the target data.

**Table 1:** Cluster Populations after Ensemble Reweighting with Segment-Averaged Target Data

Segment Length	Closed (%)	Open (%)	Semiopen (%)	Outliers (%)
1	59.1	34.4	–	6.4
5	59.5	31.0	–	9.5
10	58.9	26.6	–	14.6
15	59.1	24.9	–	16.0
20	59.5	21.0	15.6	3.9
50	61.2	10.3	22.8	5.7

Populations are measured as percentage by weight of the total ensemble. Predicted deuterated fractions from the reweighted ensembles fit the target data with *MSD* = 10^−6^. The data for segment length = 1 represent a reweighting with residue-resolved target data.

The data obtained so far assume relatively similar (60:40) populations of the open and closed states, but cases in which the ratio of states is more imbalanced can be easily envisaged. We therefore carried out additional tests with varying populations of the two states for target data consisting of 10-residue peptides. Encouragingly, HDXer was able to correctly identify trends in the target ensemble, even with low (5%) populations of either closed or open states (Fig. S7).

Even at low levels of amide resolution, the target data up to this point covered the entire length of the protein. Loss of sequence coverage increases the degeneracy of the structural information present in HDX-MS data. We therefore investigated the effects of reducing coverage using the 10-residue-long segment data set analyzed earlier, for which reweighting at 100% sequence coverage resulted in cluster populations of 58.9 and 26.6% for the closed and open states, respectively (Fig. 4 *B*; Table 1).

**Figure 5:**
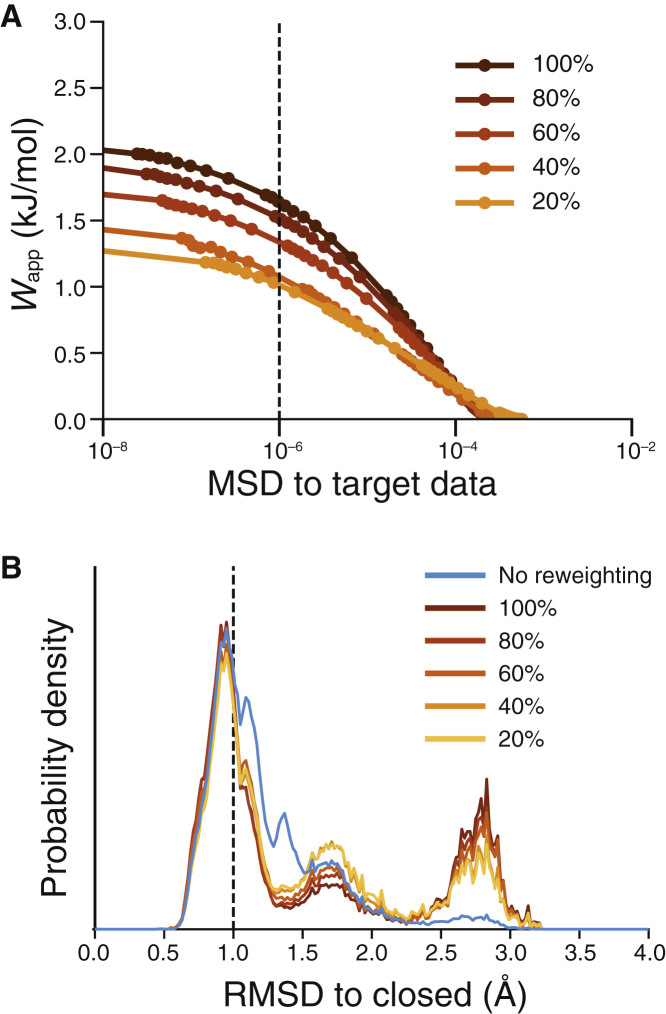
Effects of reduced sequence coverage on ensemble reweighting. (*A*) Decision plot and (*B*) RMSD probability distributions after reweighting with reduced sequence coverage in the target HDX-MS data, using target data with 10-residue segments, are shown. See legend to Fig. 4 for more details. In both panels, data from reweighting performed with full coverage are shown in dark brown, and the data obtained by decreasing sequence coverage lengths are shown using gradual color variation from light brown to orange to yellow.

As expected, gradual degradation of the sequence coverage also reduced the value of *W*_app_ required to match the target data, e.g., with *MSD* = 10^−6^ (Fig. 5 *A*), for the same reasons discussed above for increasing peptide lengths. The effect in terms of structural interpretation was also similar: reducing coverage incorrectly increased the contribution of semiopen states relative to 100% coverage (Fig. 5 *B*) and was again correlated with increasing changes to the optimized model parameters. These effects were particularly marked when the coverage was ≤40% (Table 2). Nevertheless, parameter changes remained small, up to *β*_C_ ± 1.4% and *β*_H_ ± 7.8%, after reweighting with ≥40% sequence coverage.

**Table 2:** Cluster Populations after Ensemble Refinement with Target Data Covering Smaller Proportions of the Protein

Coverage (%)	Closed (%)	Open (%)	Semiopen (%)	Outliers (%)
100	58.9	26.6	–	14.6
80	58.8	23.9	13.5	3.8
60	56.6	22.2	17.5	3.7
40	55.7	17.4	22.4	4.5
20	55.5	16.2	23.6	4.8

Populations are measured as percentage by weight of the total ensemble. Predicted deuterated fractions from the final ensembles fit the target data with *MSD* = 10^−6^. Peptide segments were 10 residues long, so the data presented for 100% coverage represent the same final ensemble as segment length = 10 in Table 1.

It is perhaps surprising that even at 20% coverage, HDXer produced a 10-fold enrichment of the population of the open state, i.e., in qualitative agreement with the target data. Inspection of the peptides included in this set (Fig. S2) shows that at least one peptide spanning the *α*9 helix was included at all coverage levels. Because the conformational change in helix *α*9 correlates strongly with the open-to-closed transition, peptides in this helix likely include crucial target observables that allow our method to correctly discern between states of TeaA. In actual HDX experiments, this correlation might not exist for any one peptide fragment among those available, in which case 20% coverage would not likely be sufficient to derive a clear interpretation. Overall, therefore, our results suggest that although low sequence coverage does not preclude ensemble reweighting, HDX-MS data at high coverage are likely to be substantially advantageous for HDXer applications.

### HDXer with noisy target data

**Figure 6:**
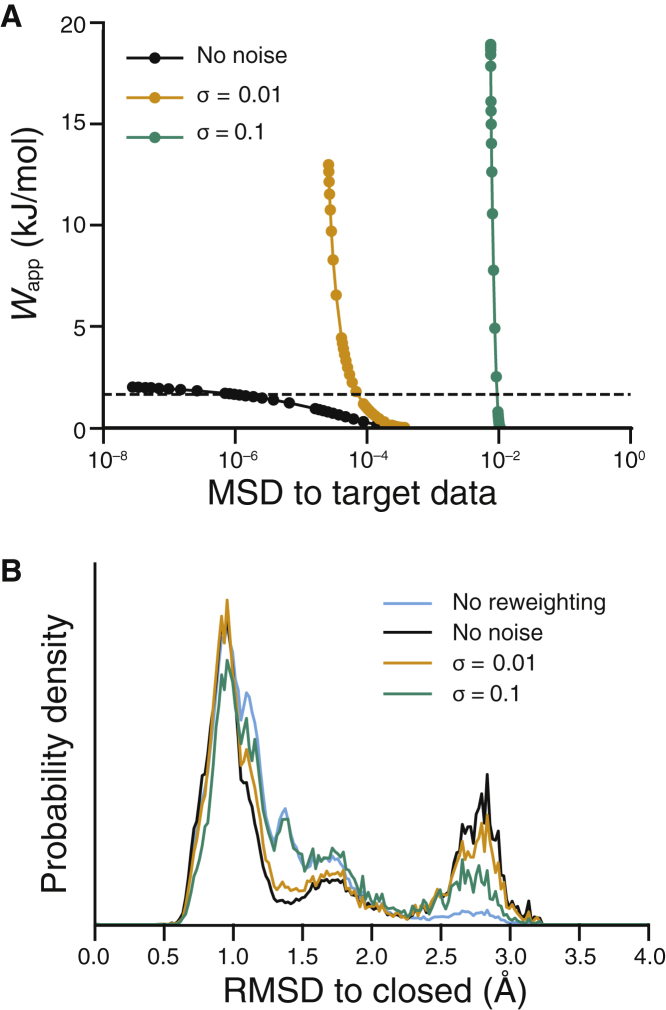
Effects of experimental noise on ensemble reweighting. (*A*) Decision plot and (*B*) RMSD probability distributions after reweighting with noise added to target data are shown. In both panels, data from reweighting performed without noise added to the target data are shown in black, while orange and teal represent results from reweighting with added noise of increasing magnitude. RMSD distributions are compared after reweighting with *W*_app_ = 1.64 kJ mol^−1^, indicated by the dashed line in (*A*). The unbiased RMSD distribution is shown in cyan.

The data so far suggest that the ability of our method to identify open and closed states from the initial sample of TeaA, based only on similarity with target HDX data, does not critically depend on peptide segment length, nor does it require complete coverage. However, all test cases so far have assumed that the target HDX data are perfectly precise, with zero random uncertainty, which is obviously not reflective of experimentally determined data. To evaluate the ability of HDXer to reweight noisy data, we added random noise of standard deviation *σ* to each target data point in the set corresponding to 100% protein coverage and 10-residue peptide segments. As expected, the accuracy of the predicted HDX-MS data after ensemble reweighting depended on the level of noise incorporated in the target data (Fig. 6 *A*). When targeting data containing Gaussian random error with *σ* = 0.01 (deuterated fraction units), a larger apparent bias (*W*_app_) was required to fit the final ensemble with *MSD* ≤ 10^−4^, compared to the experiments in which the target data were noise-free. Target data generated with *σ* = 0.1 could not be fitted with *MSD* ≤ 10^−2^, even after applying high levels of apparent bias, clearly demonstrating overfitting. Nevertheless, the final structural ensembles, evaluated at an equivalent level of *W*_app_ after reweighting with each data set, were still substantially modified from their initial distributions (Fig. 6 *B*).

In fact, with a small value of noise in the target data (*σ* = 0.01) the final ensemble showed very similar structural features to the ensemble obtained with noise-free data. When targeting data with higher noise levels (*σ* = 0.1), the final ensemble deviated more substantially from the ensemble obtained with noise-free data. In particular, HDXer was incapable of substantially downweighting semiopen structural frames (Fig. 6 *B*) when targeting the data with the most noise. For comparison, uncertainties from technical HDX-MS replicates have been estimated to be well below *σ* = 0.01, which would be equivalent to 0.1 Da error per time point per 10-residue peptide (61). Larger errors may arise from differences in experimental protocol and biological replicates (61), but our results suggest such errors may only impact the structural insights provided by HDXer if they are at least an order of magnitude larger than those typically measured between replicates. This finding provides reassurance that HDXer can provide structurally useful interpretations of HDX-MS data even with realistic levels of experimental uncertainty.

### HDXer of ensembles with insufficient conformational sampling

**Figure 7:**
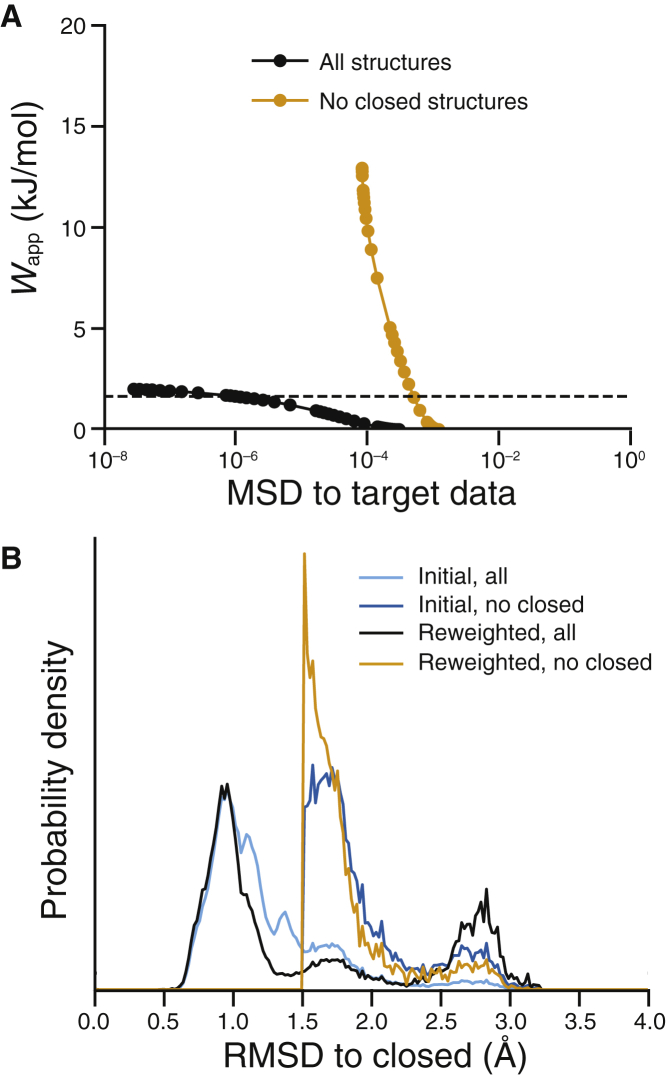
Detection of critically insufficient conformational sampling in the input ensemble to be reweighted. (*A*) Decision plot and (*B*) RMSD probability distributions after reweighting are shown. In both panels, data were obtained from reweighting performed with an initial ensemble lacking closed conformations (*orange*) or with the complete initial ensemble (*black*). For comparison, the distribution of the complete reference ensemble without reweighting is shown for the full ensemble containing all structures (*cyan*) and for the ensemble without structures of the closed conformation (*blue*). RMSD distributions are compared after reweighting with *W*_app_ = 1.64 kJ mol^−1^, denoted by the dashed line in (*A*).

In addition to potentially uninformative or noisy data, it is entirely possible that the initial ensemble being reweighted does not contain any of the structural states reflected by the HDX-MS measurements being targeted. In the case of MD sampling, this situation might arise because of sampling-time inadequacies or force-field discrepancies. For the TeaA system, this situation can be exemplified by removing all the closed-state conformations from the reference ensemble before applying HDXer exactly as before (see Methods). The resultant decision plot (Fig. 7 *A*) clearly shows that the fit could not be improved beyond an agreement of *MSD* ≈ 10^−4^, in contrast to the fits with *MSD* < 10^−7^ attained when closed-state structures were present in the reference ensemble. Concomitant with the decrease in *MSD* was a rapid increase in the apparent work required (*W*_app_ > 10 kJ mol^−1^), indicating poor overlap between the reference and reweighted ensembles, i.e., only a handful of configurations have predicted HDX values in agreement with the target data. This interpretation is supported by the distribution of structures after reweighting, which predominantly consists of semiopen states that must be only partially representative of the closed-state HDX data (Fig. 7 *B*). Encountering such a characteristic decision plot and structural distribution when using experimental data would motivate the use of enhanced-sampling methods to improve the reference pool of structures (62, 63, 64, 65).

### HDXer with inaccuracies in the empirical model

In the aforementioned data, the phenomenological model of Best and Vendruscolo used to translate structural frames into protection factors is assumed to be perfectly accurate, with constant values of the empirical scaling factors *β*_H_ and *β*_C_ used throughout the protein. In particular, the target HDX data were obtained using the values *β*_H_ = 2.0 and *β*_C_ = 0.35 that were identified based on the original parameterization (33). However, the optimum values of these scaling factors might vary between proteins or within the protein environment. Revisiting the target HDX data set and systematically varying the values of *β*_H_ and *β*_C_ indicated that the predicted HDX levels themselves were not sensitive to small changes in *β* parameters (Fig. S8). However, this analysis also illustrated that *β*_H_ and *β*_C_ should be inversely related: any perturbations away from an inverse relationship resulted in large discrepancies relative to the initial predicted HDX calculated with *β*_H_ = 2.0 and *β*_C_ = 0.35. These findings are broadly consistent with prior observations (33).

By treating *β*_H_ and *β*_C_ as additional (“nuisance”) parameters during the reweighting of *λ*_*i*_, as has been done here, any systematic inaccuracy of the model parameters across the whole protein should be automatically reduced. However, if deuteration in different regions of the protein is best described by different model parameters, the nuisance parameter optimization will result in an imprecise, “averaged” model that may be inappropriate in individual protein regions. To analyze the potential effects of model inaccuracy in a structural context, we perturbed the target data set by generating artificial HDX-MS data using different *β*_H_ and *β*_C_ parameters for residues in the *α*9 helix. Specifically, values of *β*_H_ and *β*_C_ that were either consistent or inconsistent with their observed inverse relationship (Fig. S8) were used to generate “low-error” and “high-error” target data sets, respectively.

**Figure 8:**
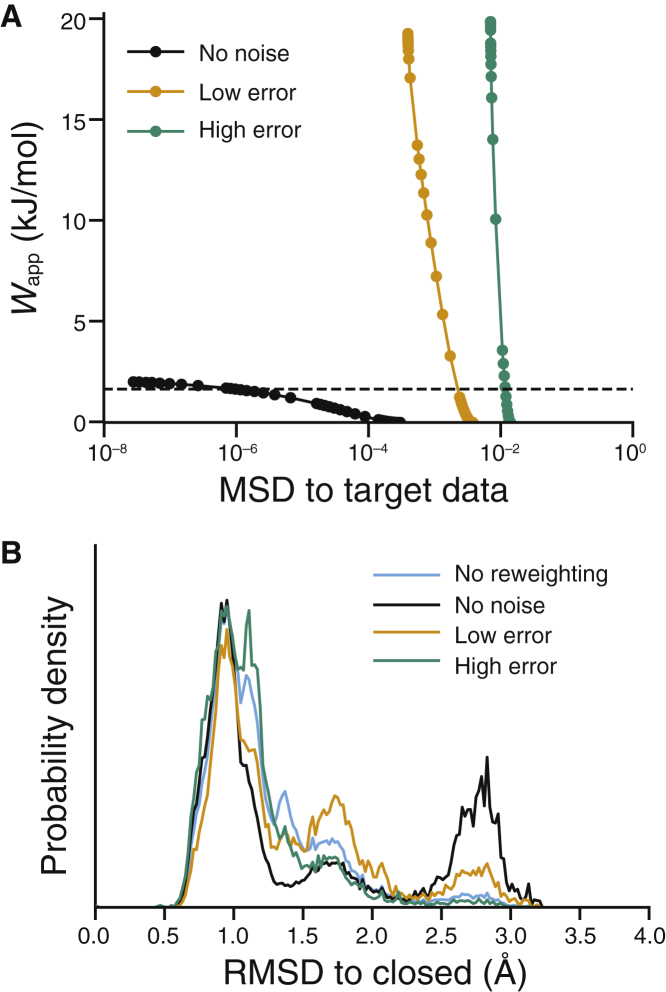
Effect of variations in the HDX model on ensemble reweighting. (*A*) Decision plot and (*B*) RMSD probability distributions after reweighting are shown. In both panels, data were obtained from reweighting to target data sets created using mixtures of *β*_H_ and *β*_C_ parameters designed to correspond with low-error (*orange*) and high-error (*teal*) conditions, alongside target sets obtained either with the default parameters (*black*) or without reweighting (*cyan*). RMSD distributions are compared after reweighting with *W*_app_ = 1.64 kJ mol^−1^, denoted by the dashed line in (*A*).

The introduction of errors in the *β* parameters was detrimental to the ability of HDXer to fit a final ensemble with moderate agreement (*MSD* < 10^−4^) to either target data set (Fig. 8 *A*), demonstrating the importance of these parameters for reweighting, at least quantitatively. Nevertheless, the structural distributions obtained from this optimization differed significantly depending on the magnitude of the perturbation to the parameters. Specifically, compared at equal values of *W*_app_, reweighting to the low-error data set resulted in a final ensemble with upweighted populations of both semiopen and open frames, whereas reweighting to the high-error target data was unable to recreate the target TeaA structural distribution (Fig. 8 *B*). The effects of the low-error parameter set are similar to the trends observed when reweighting to data sets degraded by increasing peptide length (Fig. 4 *B*) or by reducing sequence coverage (Fig. 5 *B*). In both cases, the final *β*_H_ and *β*_C_ parameters, after optimization during the HDXer reweighting procedure, differed from the values used for either the *α*9 helix or the remainder of the protein (Table S1). These results suggest that if different regions of the protein are best described by fundamentally different models, perhaps reflective of distinct exchange mechanisms, the use of a single set of averaged *β*_H_ and *β*_C_ parameters may not produce informative structural ensembles. On the other hand, HDXer can be expected to provide structurally useful information when applied to experimental HDX-MS data that are uniformly well described by a given model or in which the HDX prediction model is only slightly incorrect, for example, in which exchange is well described by *β*_H_ and *β*_C_ parameters that follow the inverse relationship of the original Best and Vendruscolo model.

### Conformational ensembles of a membrane transport protein identified by HDXer

The tests presented so far have been based on artificial HDX data, which allowed us to evaluate how the different kinds of plausible uncertainty in the input data impact the outcome of our ensemble reweighting for an assumed model of the protection factor. It is, however, reasonable to ask how HDXer might perform when applied to true experimental data. To evaluate HDXer in the context of a real-life biological question, we applied this method to experimental HDX-MS data obtained for the bacterial amino acid transporter LeuT. The structure and function of LeuT has been well studied because it serves as a prototype of a wide variety of membrane transport proteins that share its fold. The functional mechanisms of these proteins require that they alternate between states in which substrate binding sites are exposed to either the outside or the inside of the cell; these conformations are referred to as outward- and inward-facing states, respectively. Transport proteins like LeuT are therefore inherently dynamic; however, their conformational preferences can be biased by specific environmental conditions, ligands, and/or point mutations. For example, an earlier study on LeuT reconstituted into lipid nanodiscs suggested that the population of inward-facing conformations is greatly amplified by the Y268A mutation as compared to the WT protein (16). To assess whether application of HDXer would validate or refute this conclusion, we used MD simulations to generate a conformational ensemble containing a mixture of inward- and outward-facing LeuT in equal populations. This ensemble was then reweighted separately so as to reproduce HDX-MS data reported for either WT or Y268A.

**Figure 9:**
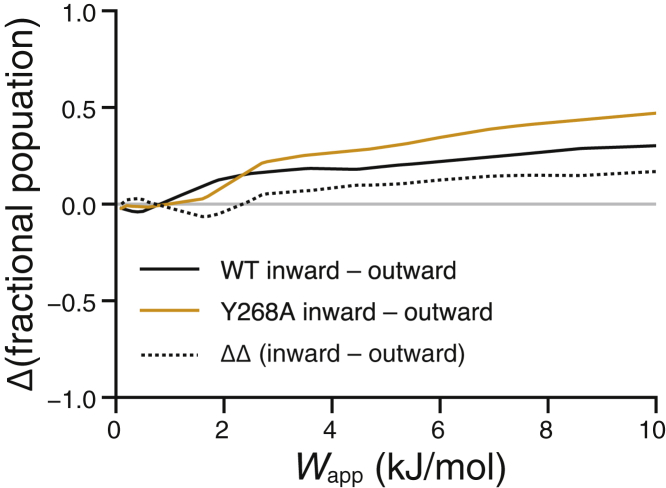
Application of HDXer to experimentally determined HDX-MS data for LeuT. A mixed reference ensemble consisting of both outward- and inward-facing LeuT structures was fitted separately to either WT LeuT (*solid black line*) or Y268A mutant HDX-MS data (*solid orange line*). The difference between the WT and mutant data is shown as a dotted line.

After reweighting, the relative population of inward- and outward-facing LeuT conformations was calculated for each set of target HDX-MS data and quantified as the excess population of inward-facing states (i.e., inward population − outward population). Application of HDXer to the Y268A target experimental data resulted in a greater excess of inward-facing conformations in the final structural ensemble, consistent with the conclusions of the original HDX-MS study (Fig. 9). Importantly, the proportion of inward-facing frames increased with *W*_app_, suggesting that improved agreement with Y268A HDX-MS data was predominantly driven by the selection of a larger population of inward-facing conformations in the final ensemble.

Available crystal structures and biophysical studies of LeuT with Förster resonance energy transfer (FRET) and double electron electron resonance (DEER) also support the observation that the Y268A mutation shifts the transporter ensemble toward inward-facing populations (66, 67, 68). The absolute populations of inward- and outward-facing states have not been reliably determined, however, and are liable to be affected by experimental differences such as the lipid or detergent environment or ionic conditions. Our interpretation of the LeuT HDX-MS data is therefore limited to identifying the trend in population shift upon Y268A mutation in lipid nanodiscs. In this context, we have established that HDXer correctly discriminates the shift toward more inward-facing states associated with the mutation.

Overall, our findings underscore the potential of the proposed method to generate structure-based interpretations of experimentally determined HDX-MS data that are not only quantitative and objective but also mechanistically informative, even when the target data are imperfect.

## Discussion

Broad applicability and label-free sample preparation have made HDX-MS an increasingly attractive biophysical technique to study global biomolecular structure and dynamics under native conditions, as demonstrated by the variety of reported applications on both globular and membrane proteins, as well as frequently updated reviews (3,4,69). The major challenge, however, has been to objectively translate the HDX data into structural information so as to be able to formulate conclusive mechanistic insights. The methodology introduced here, named HDXer, is intended to facilitate this structural interpretation. In this approach, a distribution of conformations in a pre-existing ensemble is reweighted post hoc so that calculated ensemble-averaged deuteration levels match a given set of target data. Further analysis of the resulting reweighted ensemble (for example, through clustering) thus provides the desired structural interpretation of the inputted HDX data.

As noted, the overall performance of the HDXer method was assessed on artificially-generated target data. Two factors motivated this deliberate choice. First, we aimed to focus our evaluation on the reweighting method itself, leaving aside other factors that contribute to the prediction of HDX data. By using the same empirical model of *P*_*i*_ (Eq. 1) and EX2-like kinetics, both in the generation of the artificial HDX data and in the calculation of weights (Eq. 4), we ensured that potential inaccuracies in this empirical model did not influence our assessment. Similarly, by using a pre-existing configurational ensemble with a predefined population of states to generate the artificial data, we ensured that there was a correct answer against which our methodology could be evaluated.

The second advantage of artificial data is that it can be systematically degraded in ways that reflect the limitations of actual measurements so as to judge the usability of the technique for structure determination. Indeed, HDX-MS studies vary greatly in terms of the level of peptide coverage and redundancy, and a priori there is no guarantee that an observed set of peptides will contain sufficient information to allow a clear structural interpretation. Our method performs optimally the better the coverage and resolution of the data, as one should expect. However, it is worth noting and is also very promising that even with incomplete sequence coverage or lengthy peptide segments, well beyond those typically attained in well-optimized HDX-MS experiments, our reweighting method can qualitatively identify the major conformational states contributing to the target set (Tables 1 and 2). Furthermore, the identification of relevant conformational states is consistent upon addition of moderate levels of artificial noise to the target data set. These observations, together with the encouraging results obtained for LeuT, lead us to conclude that HDXer will successfully provide structural insights when used to interpret experimentally determined data exhibiting typical coverage and noise.

Notwithstanding these reasons for optimism, it should be noted that the ability of this or any other computational method to facilitate the interpretation of measured HDX data ultimately depends on the fidelity of the empirical model used to calculate the residue protection factors, *P*_*i*_, for a given structural snapshot. Indeed, to date no HDX prediction model has yet been shown to be uniformly accurate across different biomolecular systems (70, 71, 72). In the current HDXer implementation, which predicts *P*_*i*_ using the Best and Vendruscolo forward model (Eq. 1), the structural correlate of the data is the folded protein rather than exchange-competent protein conformations. Therefore, this model is well suited to applications with ensembles generated by, for example, microsecond-timescale MD simulations. Note, however, that with sufficient sampling, HDXer may also be applied with alternative forward models that explicitly define and explore exchange-competent states (34).

Our results indicate that the Best and Vendruscolo model is sensitive to large conformational changes and assigns similar deuterium exchange levels to structurally correlated frames (Fig. 3), which are positive features that are well suited to ensemble reweighting. Moreover, our controlled evaluations of HDXer with model errors incorporated into the target data suggest that reweighting with this model can provide structural insights even when applied to experimentally determined data. On the other hand, our evaluation also makes it clear, reassuringly, that a reweighting method will not be practically useful if the reference ensemble does not include the conformational states present in the target HDX-MS data.

Given the different sources of potential error, it is crucial to be able to assess the reweighting process in absolute terms, i.e., to discern when the optimal solution is less than realistic. The HDXer method is equipped to do so, specifically through the calculation of the *W*_app_ required to achieve a given *MSD*. *W*_app_ and other metrics of reweighting robustness such as the Kish effective sample size (51,73) may be used to identify situations in which the reweighting results are liable to overfitting. Along a similar vein, *W*_app_ and *MSD* may also be used as metrics to rank results obtained using alternative empirical protection factor models or alternative reference ensembles so as to evaluate and improve their accuracy. The framework provided by HDXer to assess these potential sources of error is a key advantage in applications with experimental data.

Finally, the HDXer method could be straightforwardly applied to cross validate the HDX data itself (Supporting Materials and Methods). Deuteration levels measured at different time points could be separated into training and validation sets, and inconsistencies in the resultant reweighted ensembles may reveal sources of experimental error. However, as has been extensively discussed for other ensemble refinement methods (50,51,57,58,73), disentangling the exact sources of error in a given set of reweighting results is a challenging proposition and likely to require comparison and cross validation with multiple reference ensembles and experimental data sets.

On a technical note, it is worth underscoring that in contrast to the canonical maximum-entropy reweighting approach, which enforces exact agreement with an experimental observable, we use a parameter *γ* to control the degree of fitness to the target to account for potential uncertainties in the measurements. Consequently, HDXer shares some of the theoretical underpinnings of Bayesian approaches used to optimally recreate experimental observables, either through ensemble reweighting or on-the-fly biased sampling (50,57,74). We would argue, however, that biased sampling might not be an appropriate strategy to interpret HDX data, given the empirical nature of HDX prediction models and their imperfect correlation with experiment (70, 71, 72) and, more generally, our incomplete understanding of the structural determinants of exchange across different biomolecular systems. Thus, post hoc reweighting seems the most effective approach at this time.

In conclusion, we have developed an effective maximum-entropy-based method to derive a structural-level interpretation of HDX-MS experiments via reweighting of conformational ensembles. We anticipate that HDXer will contribute to more systematic, quantitative analyses of HDX prediction methodologies and aid studies of individual proteins and their functional mechanisms via objective structural interpretation of experimental HDX-MS measurements.

## Author Contributions

Conceptualization, all authors; methodology development, programming, analysis, and writing of original drafts, R.T.B. and F.M.; supervision and resource and funding acquisition, J.D.F.-G. and L.R.F.; visualization of data, R.T.B., F.M., and L.R.F.; writing—review and editing, all authors; project administration, L.R.F.

## Supporting Material

Document S1. Supporting Materials and Methods, Figs. S1–S8, and Tables S1 and S2

Document S2. Article plus Supporting Material
