# Supplementary material for: Interpretation of HDX Data by Maximum-Entropy Reweighting of Simulated Structural Ensembles
Source: Biophys J. 2020 Feb 15;118(7):1649–64. doi: 10.1016/j.bpj.2020.02.005 (PMC7136279; doi:10.1016/j.bpj.2020.02.005)
Supplement: Document S1. Supporting Materials and Methods, Figs. S1–S8, and Tables S1 and S2 [file mmc1.pdf]

Biophysical Journal, Volume *118*

Supplemental Information

Interpretation of HDX Data by Maximum-Entropy Reweighting of Simulated Structural Ensembles

Richard T. Bradshaw, Fabrizio Marinelli, José D. Faraldo-Gómez, and Lucy R. Forrest

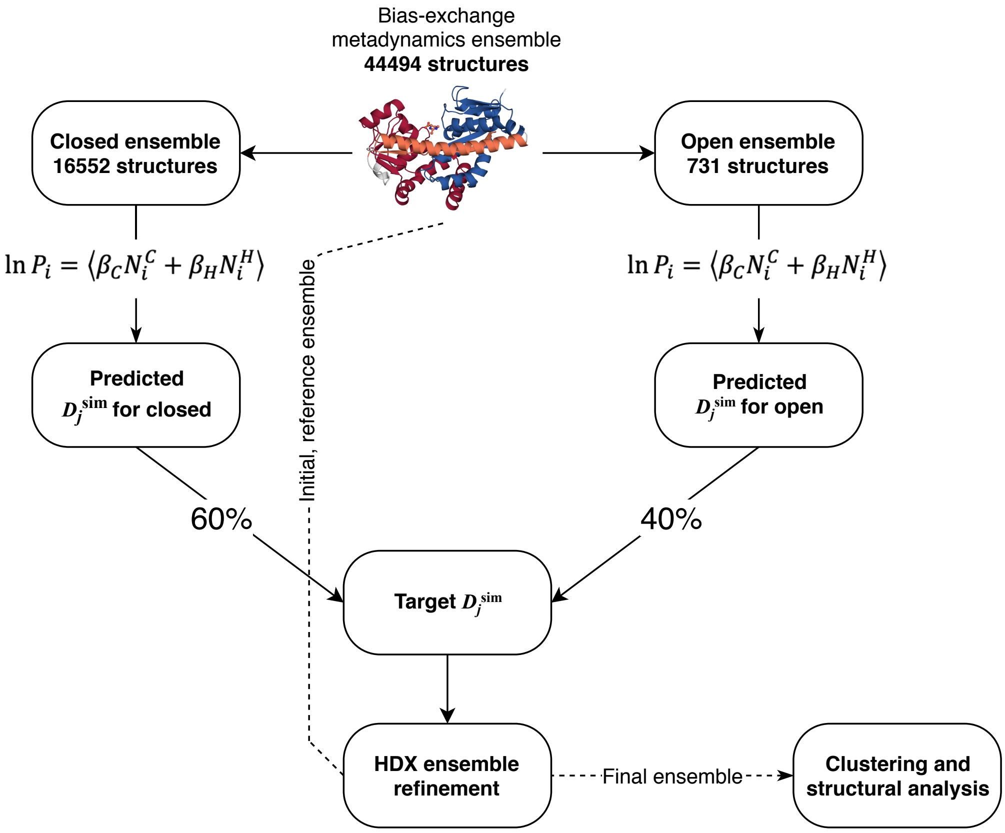

Figure S1 – Structural ensembles and target data used for HDX ensemble refinement testing. Artificial HDX data (*solid arrows*) were generated for each exchangeable amide for a mixed ensemble corresponding to 60% closed and 40% open TeaA. These HDX data were used as the target for ensemble refinement of the complete test ensemble, which includes closed, open, and decoy semi-open frames (*dashed lines*).

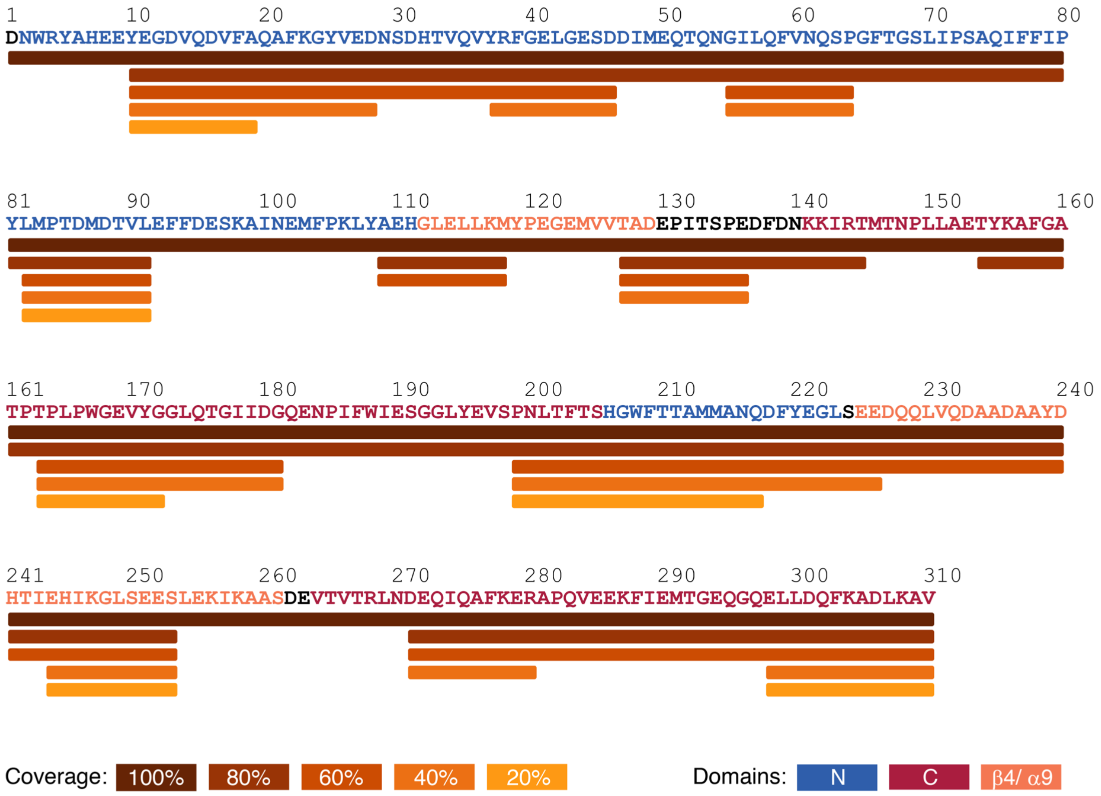

Figure S2 – Residues included in target HDX data for TeaA with reduced levels of sequence coverage. The sequence is colored according to the protein domain region as defined in Fig. 1A. Bars underneath the sequence indicate the residues included in the target data at 100% coverage (*dark brown*) and at approximately 80, 60, 40, or 20% coverage (from *light brown to orange to yellow*). See Methods section in the main text for more details.

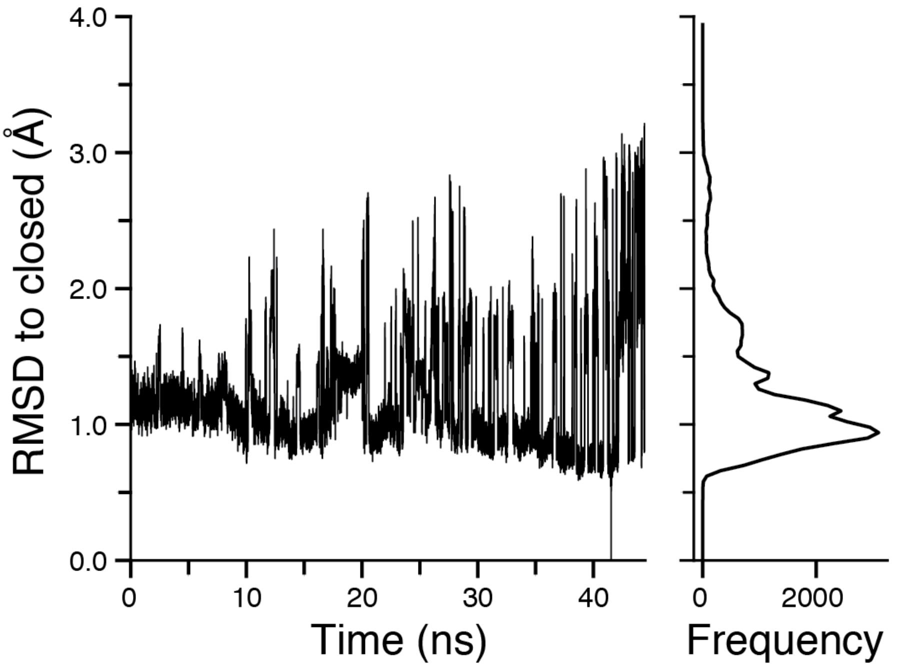

Figure S3 – Structural variability in the reference simulation of TeaA and selection of reference structures. The root mean square deviation (RMSD) of the C_α atoms from the reference closed conformation is plotted against time for the unbiased trajectory from bias-exchange metadynamics simulations performed by Marinelli & coworkers (1). Trajectory frames were taken at 1 ps intervals. The frame at 41.546 ns was arbitrarily selected as the reference closed configuration, among many frames with similarly-low RMSD from the holo structure. Visual analysis confirmed that this configuration adequately represented the two key features of closed structures, namely a small inter-lobe distance and a kinked α9 helix. The frame at 44.428 ns has the highest RMSD to the closed conformation and was therefore selected as the reference open configuration. The distribution of the RMSD values to the closed structure is shown in the panel on the right.

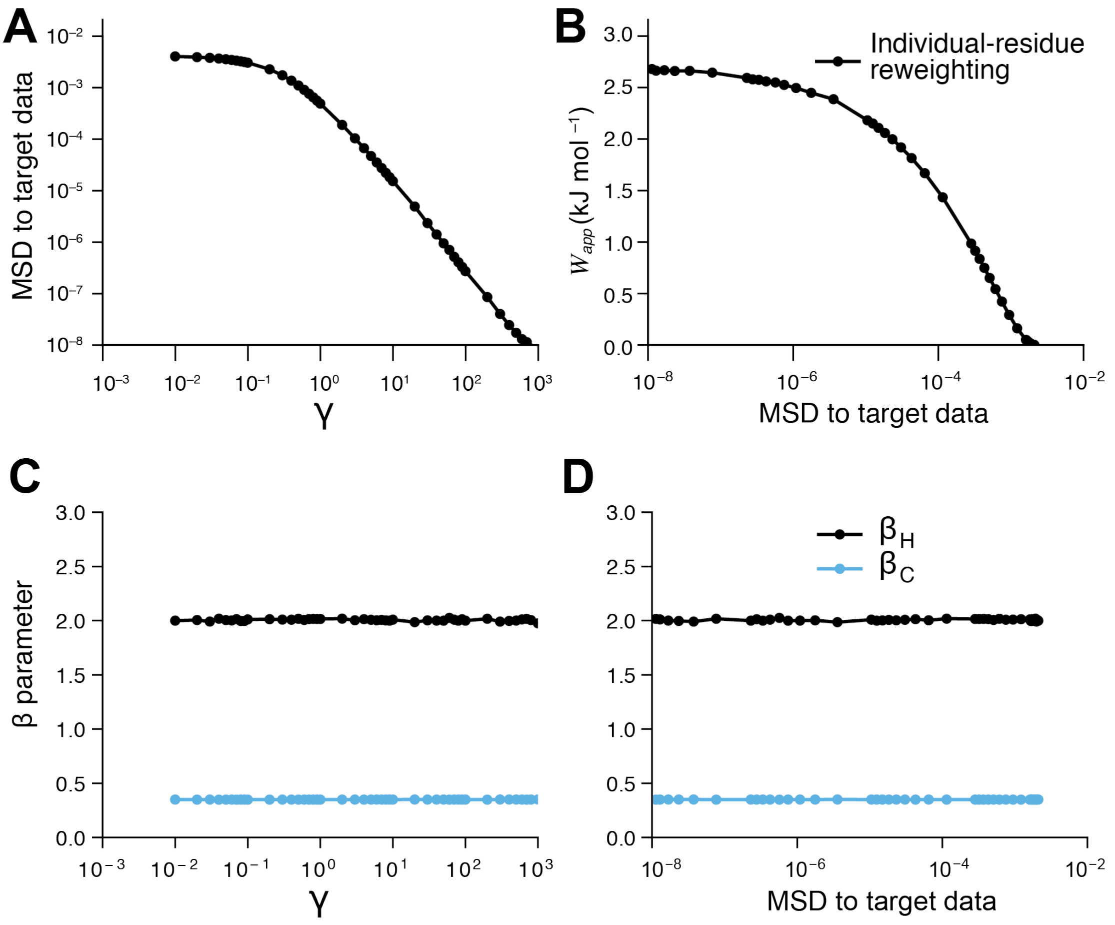

Figure S4 – Relationship between γ, W_{app}, and the agreement with the target data during HDX reweighting analyses in which the target TeaA HDX data is resolved at the individual residue level. Circles indicate independent reweighting experiments. **(A)** The mean square deviation (MSD) between the predicted and target HDX values increases as the value of γ decreases. Little improvement in MSD is observed for values of γ below ca. 10^{-1}, suggesting that, beyond this point, the initial HDX data lies within the uncertainty distribution ρ_{err} that is defined by γ. **(B)** For the same reweighting analyses, the reduction in MSD is coupled to an increase in W_{app} until a plateau is reached for MSD values below ca. 10^{-7}. **(C)** and **(D)** Optimized values of the β_{H} (*black*) and β_{C} (*blue*) parameters after reweighting do not change as a function of γ or MSD. The target HDX-MS data was generated using β_{H} = 2.0 and β_{C} = 0.35.

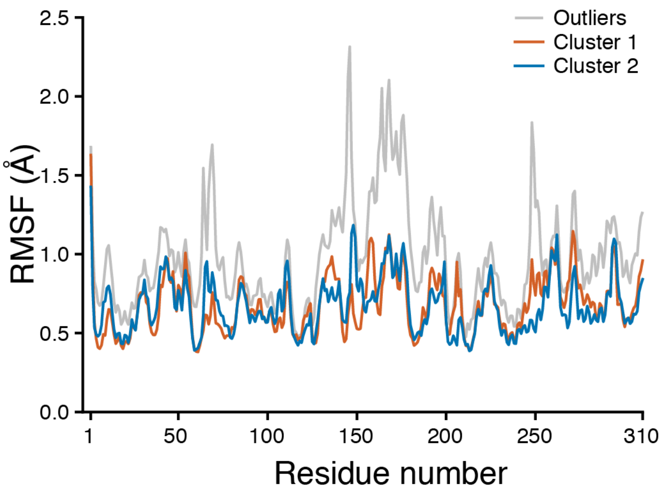

Figure S5 – Structural variance in the clustered ensembles of TeaA obtained after ensemble reweighting by HDX data resolved at the individual residue level. The backbone root mean squared fluctuation (RMSF) was averaged over each residue for all conformations in the two main clusters (*orange and cyan*), or in the outliers (*gray*).

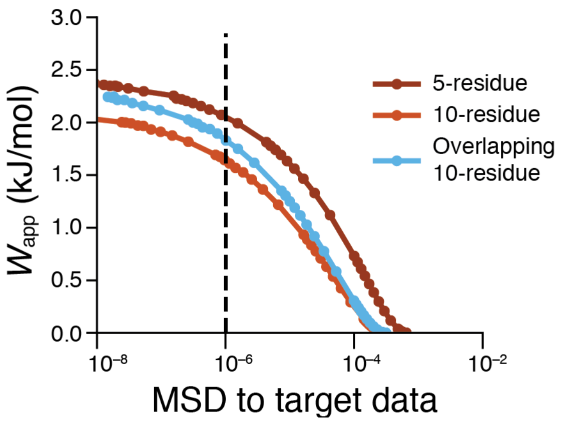

Figure S6 – Effect of overlap (i.e., redundancy) in the peptide segment residue ranges on reweighting. The decision plot shows the work applied during reweighting against the MSD of the reweighted ensemble to the target HDX data. Circles indicate independent reweighting experiments. Target data with overlapping 10-residue segments were defined such that peptides maintained 100% sequence coverage, but overlapped in approximately 5-residue intervals (e.g., residues 1-10, 6-15, 10-19, 15-24…). Reweighting using overlapping segments (*blue*) initially shows similar performance to reweighting with non-overlapping 10-residue segment data (*light brown*), but trends towards the results obtained with shorter, 5-residue, peptide segments (*dark brown*), owing to the additional information content of target data with peptide redundancy.

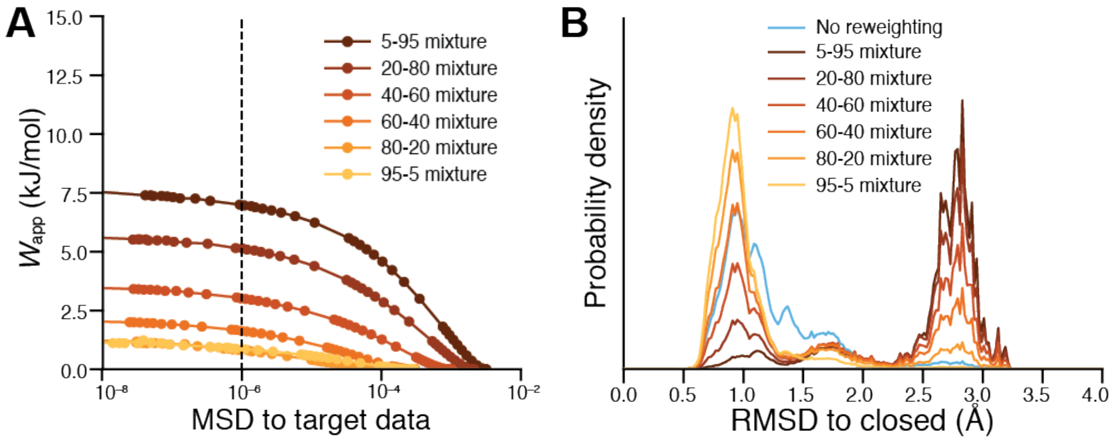

Figure S7 – Effect of targeting the HDX reweighting to ensembles with varying populations of the two major states. Target ensembles are defined as percentage mixtures of closed and open states. The 60-40 mixture is the target ensemble used in the main text. All data assume 10-residue long peptide segments. **(A)** Decision plot showing the apparent work applied during reweighting, W_{app}, against the MSD of the reweighted ensemble to the target HDX data. As the target population of closed states decreases from 80% to 5%, greater values of W_{app} are required to achieve an agreement of MSD = 10^{-6} (*dashed line*). Reweighting to mixtures with 80% and 95% closed states required approximately equal W_{app}, as the large increase in closed state population compared to the reference ensemble was balanced by a comparatively smaller increase in open state population. In all cases the reference ensemble populations were 37.2 : 1.6 : 61.2 for closed : open : decoy states. **(B)** RMSD distributions, with reference to the closed TeaA conformation, after reweighting. Trends in the target ensembles are at least qualitatively recreated across all datasets.

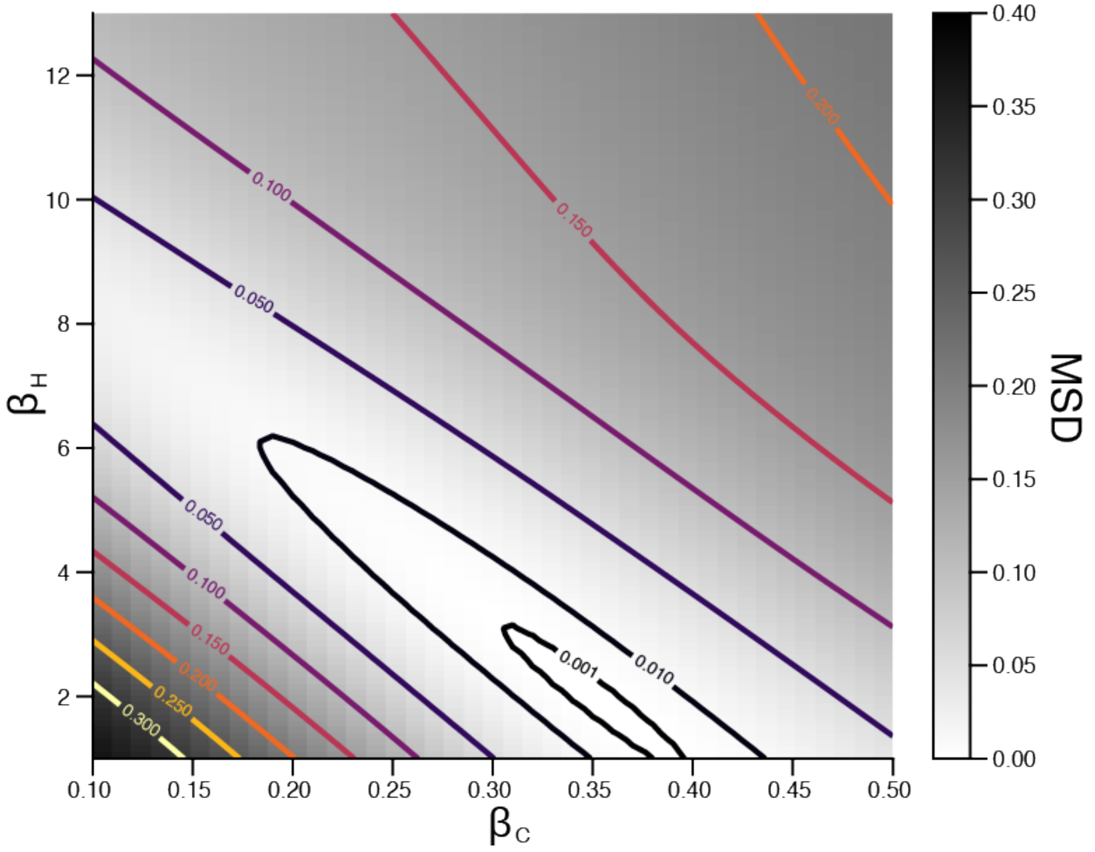

Figure S8 – Effects of varying the β parameters on predicted HDX values obtained using the Best and Vendruscolo forward model for TeaA. Contours indicate the mean square deviation (MSD) of the predicted HDX to the reference HDX values calculated with β_{H} = 2.0 and β_{C} = 0.35. The artificial TeaA HDX data generated with 100% sequence coverage in 10-residue peptide segments were used as reference HDX values.

Table S1 – HDX model parameters, β_{C} and β_{H}, after reweighting to original, low-error and high-error target datasets, with W_{app} = 1.64 kJ mol^{-1}

Dataset	*Target β_H	*Target β_C	†Observed β_H	†Observed β_C
Original	2.0	0.35	2.02	0.349
Low error	7.0	0.20	2.61	0.336
High error	2.0	0.20	1.67	0.343

* Values of β_{H} and β_{C} used to calculate target HDX-MS data for the α9 helix of TeaA; HDX-MS data for the remaining residues were calculated using the default parameters, β_{H} = 2.0 and β_{C} = 0.35. † Values of β_{H} and β_{C} obtained by optimization during the reweighting procedure.

Table S2 – Agreement of reweighted ensembles and target data for noisy and zero-noise datasets, measured as MSD. Each row reflects a cross-validation of a reweighted ensemble (trained model) against multiple target HDX data (test datasets). The accuracy of each reweighted ensemble to its own training data is shown in **bold**.

Training dataset for reweighting	Test dataset for MSD calculation					
	No noise		σ = 0.1		σ = 0.01	
	1 min	60 min	1 min	60 min	1 min	60 min
No noise	**1.02 x 10^{-6}**	**8.06 x 10^{-7}**	1.34 x 10^{-2}	7.68 x 10^{-3}	1.30 x 10^{-4}	7.71 x 10^{-5}
σ = 0.1	2.11 x 10^{-4}	3.40 x 10^{-4}	**1.25 x 10^{-2}**	**7.12 x 10^{-3}**	4.02 x 10^{-4}	4.33 x 10^{-4}
σ = 0.01	3.32 x 10^{-5}	3.32 x 10^{-5}	1.33 x 10^{-2}	7.97 x 10^{-3}	**8.73 x 10^{-5}**	**5.52 x 10^{-5}**

Text S1 – Cross-validation experiments with HDXer reweighted ensembles

Overfitting is an unwanted, but common, pitfall of methodologies designed to construct or refine structural ensembles based on limited-resolution data. We therefore evaluated the potential for overfitting in the context of HDXer. In the article enclosed we highlighted how the apparent work (W_{app}) as a function of the MSD provides an effective descriptor of the reweighting process; when this apparent work increases in magnitude with no significant improvement in MSD, overfitting is likely the cause. Nevertheless, it is unlikely that monitoring of a single metric will unambiguously preclude overfitting, and thus complementary approaches, such as cross validation experiments, are desirable. To illustrate possible applications of HDXer for cross-validation, we first defined training and test HDX-MS datasets. Specifically, we treated the reweighted ensemble as if it were a trained model with a given level of accuracy (e.g., MSD = 1 x 10^{-6}) to the target HDX data that were used for the training (reweighting) of the ensemble. In the absence of overfitting, a statistically-robust reweighted ensemble should have similar accuracy when compared to the training set data as to test data that were *not* included in the reweighting process, but were sampled from the same underlying distribution. Our first cross-validation test investigated the reweighting performed with the data comprised of 10-residue segments and with 100% coverage (**Fig. 4**). We generated new test HDX-MS data at two timepoints (5 and 30 min) that were *not* used in the HDXer reweighting, which was instead "trained" with HDX data at 0.167, 1, 10, 60, and 120 min timepoints. The reweighted ensemble had similar agreement with the test HDX dataset (MSD = 3.01 x 10^{-6} and 1.04 x 10^{-6} for the 5- and 30-minute timepoints, respectively) as with the training dataset (MSD = 1 x 10^{-6}). This result reassuringly suggests that the final ensemble had not been overfitted in this case.

With HDX-MS experiments, multiple test datasets can be easily acquired, for example from multiple independent repeat samples and multiple independent deuteration timepoints. A robustly-reweighted ensemble should agree similarly to all test data that are sampled from the same protein structural distribution, provided that the level of uncertainty of the test data is similar to that of the training data. Conversely, if test and training data feature different levels of uncertainty, in a robustly-reweighted ensemble the accuracy in reproducing each data set must be comparable with the relative error level. Our second cross-validation therefore involved test HDX datasets with a *different level of experimental noise* to the training sets included in the reweighting. Specifically, using the final ensembles obtained after reweighting to noisy target data (**Fig. 6B**), we calculated the MSD to the zero-noise target data at the 1- and 60-min timepoints. *Vice-versa*, using the final ensemble after reweighting to

zero-noise target data, we also calculated the MSD to the noisy target data at the 1- and 60-min timepoints. In each cross-validation case, the reweighted ensembles exhibit different levels of agreement to the test datasets than to the training dataset included in the HDXer reweighting. Nonetheless, these results are fully compatible with the noise level in each data set, underscoring that the reweighted ensembles were generated to a level of precision consistent with the uncertainty present in the target data (**Table S2**).

These tests demonstrate how cross-validation may be used to assess whether reweighted ensembles suffer from overfitting. Of course, this application is easily characterized here, in part owing to our use of artificial datasets with controlled sources of error. In practice, identifying potential sources of overfitting (e.g., experimental datapoints with differing uncertainty levels, simulated ensembles that do not adequately describe the variance of the experimental data, etc.) might require extensive investigation. Here, we limit ourselves to highlighting this potential use of HDX-MS data, and we propose that similar approaches will be advantageous when applied to real experimental target data that require thorough cross-validation of reweighted structural ensembles.

Movie S1 – Artificially-generated morph between the closed and open representative structures of TeaA. TeaA is shown in cartoon representation (*wheat*), and the ectoine substrate from the closed configuration is shown in ball and stick representation (*peach*).

References for Supporting Materials

1. Marinelli, F., S.I. Kuhlmann, E. Grell, H.-J. Kunte, C. Ziegler, and J.D. Faraldo-Gómez. 2011. Evidence for an allosteric mechanism of substrate release from membrane-transporter accessory binding proteins. Proc. Natl. Acad. Sci. U. S. A. 108: E1285–E1292.
